# Unraveling Predominantly Inattentive ADHD (ADHD-PI): Insights from Proteomic Analysis of the Striatum of Thyroid Hormone-Responsive Protein (THRSP)–Overexpressing Mice

**DOI:** 10.1007/s12035-025-05031-z

**Published:** 2025-06-10

**Authors:** Raly James Perez Custodio, Leandro Val Sayson, Ara Cho, Hyeryeon Jung, Darlene Mae Ortiz, Hyun Jun Lee, Emad Alyan, Edmund Wascher, Stephan Getzmann, Mikyung Kim, Kyeong‑Man Kim, Eugene C. Yi, Hee Jin Kim, Jae Hoon Cheong

**Affiliations:** 1 Networking Group Aging, Department of Ergonomics, Leibniz Research Centre for Working Environment and Human Factors at TU Dortmund (IfADo), Ardeystrasse 67, 44139 Dortmund, Germany; 2 Department of Pharmacy, Uimyung Research Institute for Neuroscience, Sahmyook University, 815 Hwarangro, Nowon-gu, Seoul, 01795 Republic of Korea; 3 Graduate School of Convergence Science and Technology and College of Medicine, Department of Molecular Medicine and Biopharmaceutical Sciences, Seoul National University, 103 Daehak-ro, Jongno-gu, Seoul, 03080 Republic of Korea; 4 College of Pharmacy, Dongduk Women’s University, 60 Hwarang-ro, Seongbuk-gu, Seoul, 02748 Republic of Korea; 5 Experimental Ergonomics, Department of Ergonomics, Leibniz Research Centre for Working Environment and Human Factors at TU Dortmund (IfADo), Ardeystrasse 67, 44139 Dortmund, Germany; 6 Department of Chemistry & Life Science, Sahmyook University, 815 Hwarang-ro, Nowon-gu, Seoul, 01795 Republic of Korea; 7 Department of Pharmacology, College of Pharmacy, Chonnam National University, 77 Yongbong-ro, Buk-gu, Gwangju, 61186 Republic of Korea; 8 Institute for New Drug Development, College of Pharmacy, Jeonbuk National University, 567 Baekje-daero, Deokjin-gu, Jeonju, 54896 Republic of Korea; 9 Addiction Research Group, Schulich School of Medicine & Dentistry, University of Western Ontario, London, N6A 5C1 Canada

**Keywords:** Adult ADHD, Thyroid hormone-responsive protein, THRSP-OE mice, Snap25, SNARE complex, Animal model

## Abstract

Attention-deficit/hyperactivity disorder, or ADHD, is a neurodevelopmental disorder with poorly understood molecular mechanisms. Recent studies have proposed that gene expression involved in regulating synaptic transmission in the striatum may play a role in ADHD pathogenesis. To explore the molecular basis of ADHD, we utilized proteomic analysis using whole striatal tissues from early adult thyroid hormone-responsive protein-overexpressing (THRSP-OE) mice, which displayed defining characteristics of predominantly inattentive ADHD (ADHD-PI). We focused on the striatal brain region due to its critical role in the regulation of attention, motivation, and reward processing. Moreover, the striatum modulates dopaminergic pathways that are known to be impaired in ADHD. Our analysis revealed an innate overexpression of Snap25 protein in THRSP-OE mice, indicating possible alterations in the SNARE protein complex and potential neurotransmitter dysregulation. Furthermore, a binding affinity study showed reduced dopamine D1 receptor binding concentrations and pronounced low dopamine levels in THRSP-OE mice. Repeated seven-day injections of methylphenidate improved the low dopamine levels, reducing the EEG theta/beta ratio in this animal model. These findings suggest new markers specific to the ADHD-PI presentation and further support the role of Snap25 dysregulation and possible SNARE protein complex alterations in ADHD-PI.

**Supplementary Information:**

The online version contains supplementary material available at 10.1007/s12035-025-05031-z.

## Introduction

Attention-deficit/hyperactivity disorder (ADHD) is a common neurodevelopmental disorder characterized by symptoms of inattention, hyperactivity, and impulsivity [1]. Despite its high prevalence, the underlying molecular mechanisms of ADHD remain poorly understood. Contemporary studies have suggested that changes in the expression of genes involved in the regulation of synaptic transmission in the striatum, a key brain region involved in attention, reward processing, and motor control, may play a role in the pathogenesis of ADHD. One such gene is synaptosomal-associated protein, 25 kDa or Snap25, which is altered in ADHD patients and animal models of ADHD [2–4], indicating a relationship between Snap25 and ADHD. Snap25 is a protein that plays a key role in regulating neurotransmitter release, and it has been linked to ADHD due to its involvement in dopamine signaling [5]. However, the exact nature of the relationship between Snap25 and ADHD is still the subject of ongoing research, and more studies are needed to understand this complex relationship fully.

In our previous study, we found that overexpressing the thyroid hormone-responsive protein (THRSP) (a.k.a. *spot14* gene) induced ADHD predominantly inattentive (PI)-like behaviors in mice [6, 7]. Interestingly, these thyroid and dopaminergic function alterations may be responsible for this behavior, as both systems are directly involved in regulating cognitive and attention behaviors [6, 8]. Since THRSP is a thyroid hormone-related protein and is highly responsive to thyroid hormone, and since thyroid hormone and dopamine share tyrosine as a common basic unit, it is possible that functional overexpression of THRSP could influence the dopaminergic system in THRSP-OE mice [9].

To significantly advance our understanding of the ADHD-PI-like behaviors identified in THRSP-OE mice and the potential mechanisms involved, we employed molecular, pharmacological, and electroencephalographic approaches. These investigations revealed that Snap25, a synaptic protein critical for neurotransmitter release and known to regulate attention and implicated in ADHD, may be involved in the observed ADHD-PI-like behaviors in early adult THRSP-OE mice. Moreover, our study demonstrated that methylphenidate administration yielded promising results in THRSP-OE mice, with notable enhancements in dopamine levels and a significant reduction in electroencephalography (EEG) theta/beta ratio (TBR) following 7 days of drug administration. These findings support the use of THRSP-OE mice as a model for adult ADHD and raise the possiblity that THRSP may be involved in the regulation of dopamine-related mechanisms relevant to the disorder.

## Materials and Methods

### Animals

The mice used in this study were developed and obtained based on our previous studies on THRSP-OE and THRSP-KO mice [6–8]. In particular, the THRSP-OE mice model evaluated here has already been identified and validated to show impairments in attention and memory with the absence of hyperactivity and impulsivity indicating its potential use as a model for ADHD predominantly inattention presentation [9]. Non-transgenic littermates were used as WT counterparts. The mice were housed under controlled temperature (22 ± 2 °C) and humidity conditions (55 ± 5%) with a 12/12-h light/dark cycle (07:00–19:00 h light). All animal care and procedures followed the Principles of Laboratory Animal Care (NIH Publication No. 85–23, revised 1985), the Animal Ethics Review Board of Sahmyook University, South Korea (SYUIACUC2023-002), and complied with the 3Rs framework and ARRIVE guidelines recommended by *Molecular Neurobiology*. Male mice were exclusively used in the experiment because of established sex differences in ADHD prevalence, and to maintain consistency with the DSM-5 criteria for ADHD symptomatology. Also, since the gene (Thrsp or THRSP) manipulated in such animal models is a thyroid hormone-responsive protein, which may impact behavior differently between males and females, the study only focused on male mice. However, future studies will take sex differences into consideration. The mice were 7 weeks old during the experiment, indicating full body growth and at early adulthood age [10], similar to our previous study [7].

### Brain Extraction

Brain samples (striatum; STR) were taken from a total of seventy-two 7-week early adult mice. The striatal tissue samples were randomly assigned and subjected to proteomic analysis (*n* = 6/group, with an additional THRSP-KO group (*n = 6*)), real-time reverse transcription polymerase chain reaction (RT-qPCR) (*n* = 6/group), receptor-binding affinity studies (*n* = 5/group), and enzyme-linked immunosorbent assay (ELISA) (*n* = 8/group for THRSP-OE and WT mice, each treated with vehicle (VEH) and methylphenidate (MPH). The THRSP-KO samples were included exclusively for the proteomic analysis to serve as supplemental comparative data. This group was not the primary focus of the study, which specifically targeted the THRSP-OE model due to its relevance to ADHD-PI-like behaviors. Therefore, all subsequent analyses and tests were conducted by comparing results between THRSP-OE and WT mice. The striatal tissue samples were snap-frozen, placed in a dry ice-filled box, and then transported to our collaborators at the Seoul National University (for proteomic studies) and Chonnam National University (for binding-affinity studies) or stored in-house at Uimyung Research Institute for Neuroscience at Sahmyook University (for RT-qPCR and ELISA) until further use.

### Proteomic Analysis

**Fig. 1:**
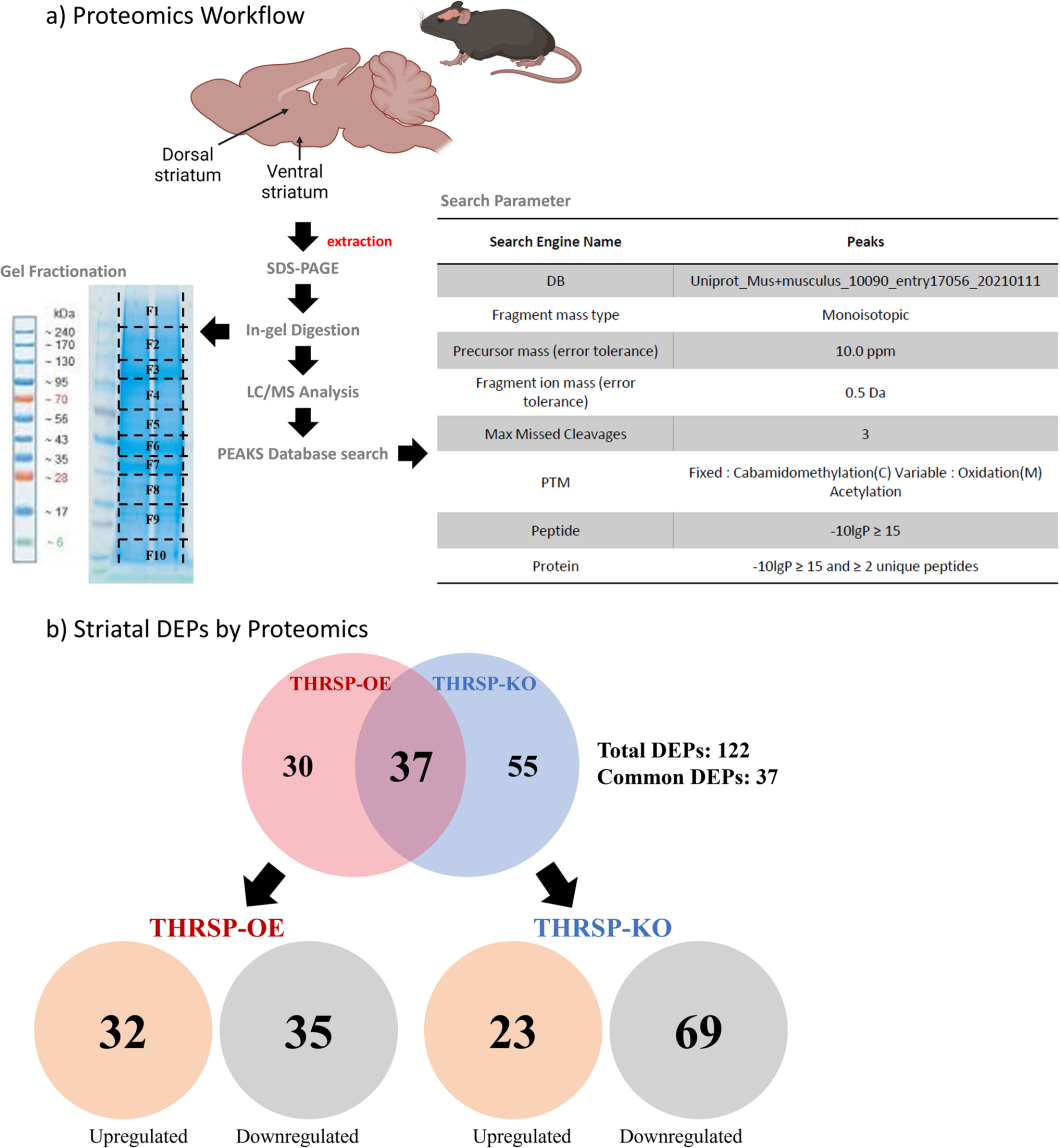
Striatal proteomics workflow in thyroid hormone-responsive protein (THRSP) transgenic early adult (7 weeks old) mice (*n* = 6/group). **a** With known ADHD-PI-like behaviors, mice striatum was subjected to subsequent proteomic analysis. **b** Summary of identified striatal DEPs in THRSP-OE and THRSP-KO relative to wild-type mice. Abbreviations: SDS-PAGE, sodium dodecyl-sulfate polyacrylamide gel electrophoresis; LC/MS, liquid chromatography–mass spectrometry; DEPs, differentially expressed proteins; THRSP, thyroid hormone-responsive protein; OE, overexpressed; KO, knockout. The images used in **a** was generated by BioRender.com

The protocol used in the proteomics studies, as shown in Fig. 1a, was based on our previous study [7]. In brief, proteins were extracted from mouse striatal brain tissue samples using a radioimmunoprecipitation assay (RIPA) buffer composed of 50 mM Tris–HCl, 150 mM NaCl, 1% NP-40, 0.5% sodium deoxycholate, and 1% SDS. The extracted proteins were then quantified by bicinchoninic acid (BCA) protein assay. Two hundred milligrams of each sample was taken and, under reducing conditions, was resolved by sodium dodecyl sulfate–polyacrylamide gel electrophoresis (SDS-PAGE), followed by Instant Blue Coomassie protein staining. Each lane was divided into ten separate segments for in-gel protein digestion. The stained gel fragments were sectioned, washed with 100 mM ammonium bicarbonate, and dehydrated with 50% acetonitrile in 25 mM ammonium bicarbonate. The reduction was performed by incubating the samples with 20 mM dithiothreitol (DTT) for 1 h at 60 °C, followed by 55 mM iodoacetamide incubation for 45 min in the dark for alkylation. After washing and dehydration with acetonitrile, the gel sections were incubated with 12.5 ng/μL trypsin in 50 mM Ammonium bicarbonate overnight and then stored at 37 °C to allow digestion. Peptide extraction was then carried out by incubation with 10% formic acid and further incubation with 50% acetonitrile in 0.1% formic acid and 80% acetonitrile in 0.1% formic acid at 37 °C. Further, the eluted peptides were dried in a SpeedVac and then stored at −20 °C until further use.

### Liquid Chromatography–Mass Spectrometry (LC–MS/MS) and Data Analysis

The methodology used for LC/MS/MS, as depicted in Fig. 1a, was based on our previous research [7]. In brief, peptides were dissolved in solvent A (0.1% formic acid) and loaded onto an analytical column. They were then separated with a linear gradient of solvent B (0.1% formic acid in 98% acetonitrile) from 5 to 35% over 95 min at a 300 nL/min flow rate. The MS spectra were recorded using a Q-Exactive Plus hybrid quadrupole-orbitrap mass spectrometer and an Ultimate 3000 high-performance liquid chromatography system. The spray voltage standard mass spectrometric condition was 2.0 kV, and the heated capillary temperature was 250 °C. Full scans were acquired in the 400–1400 m*/z* range with a resolution of 70,000 while setting the normalized collision energy to 27% with 17,500 resolutions, allowing fragmentation using high-energy collision dissociation. Data-dependent acquisition with a single survey MS scan followed by 10 MS/MS scans was performed with an exclusion time of 30 s. Using engine-based PEAKS studio, all collected MS/MS raw data were converted into mzXML files. Protein identification was carried out using the UniProt-Mus musculus database, with fragment mass tolerance of 0.8 Da and precursor mass tolerance of 10 ppm. Methionine oxidation was considered a variable amino acid modification, whereas cysteine carbamidomethylation was deemed a fixed modification. The enzyme selected for the experiment was trypsin, which allows up to two missed cleavages. The protein and peptide identifications were filtered to less than 1% false discovery rate (FDR) using a concatenated target-decoy database search strategy. The R program’s Power Law Global Error Model (PLGEM) package (version 3.4.2) was used for relative protein quantitation analysis. PLGEM controls datasets to distinguish statistically significant differentially expressed proteins (DEPs) and calculates changes in expression levels by p-value and signal-to-noise (STN) ratio. Further, the ingenuity pathway analysis was then performed using all significantly altered proteins, as shown in Fig. 1b and Tables 1 and 6. The MS proteomics data have been deposited to the ProteomeXchange Consortium via the PRoteomics IDEntifications Database (PRIDE) [11] partner repository with the dataset identifier PXD051619.

**Table 1:** The striatal DEPs analyzed from THRSP transgenic (OE and KO) mice relative to WT mice

DEPs (no.)	Gene name	Accession	Description	THRPS-OE		THRPS-KO	
				STN ratio	*p*-value	STN ratio	*p*-value
1	Dennd4c	A6H8H2	DENN domain-containing protein 4 C	1.3632	0.0058	− 1.1291	0.0128
2	Ptprs	B0 V2 N1	Receptor-type tyrosine-protein phosphatase S			− 1.0163	0.0186
3	Cttnbp2	B9EJA2	Cortactin-binding protein 2			− 1.2154	0.0072
4	Dgki	D3YWQ0	Diacylglycerol kinase iota			− 1.5173	0.0018
5	Fga	E9PV24	Fibrinogen alpha chain			1.0491	0.0211
6	Mctp1	E9PV86	Multiple C2 and transmembrane domain-containing protein 1			− 1.4272	0.0032
7	Atp2b1	G5E829	Plasma membrane calcium-transporting ATPase 1	− 1.1282	0.0128		
8	Bin1	O08539	Myc box-dependent-interacting protein 1	− 1.8097	0.0004	− 3.2773	0.0000
9	Stxbp1	O08599	Syntaxin-binding protein 1	− 1.2700	0.0057		
10	Snap23	O09044	Synaptosomal-associated protein 23	1.4764	0.0033		
11	Snca	O55042	Alpha-synuclein			− 1.2235	0.0065
12	Stx7	O70439	Syntaxin-7	1.1669	0.0135		
13	Vamp4	O70480	Vesicle-associated membrane protein 4	1.4764	0.0033		
14	Vti1b	O88384	Vesicle transport through interaction with t-SNAREs homolog 1B	1.6926	0.0013		
15	Ddc	O88533	Aromatic-L-amino-acid decarboxylase	− 3.5795	0.0000	− 3.3286	0.0000
16	Syn1	O88935	Synapsin-1	− 1.5979	0.0008	− 2.3051	0.0001
17	Prkaca	P05132	cAMP-dependent protein kinase catalytic subunit alpha	− 2.5336	0.0000	− 1.5504	0.0013
18	Kit	P05532	Mast/stem cell growth factor receptor Kit			− 1.4267	0.0032
19	Ctsl	P06797	Procathepsin L			− 1.0438	0.0163
20	Sparc	P07214	SPARC	1.7321	0.0012		
21	Itgb1	P09055	Integrin beta-1	1.2191	0.0100		
22	Calm1	P0DP26	Calmodulin-1	1.5869	0.0019		
23	Calm3	P0DP28	Calmodulin-3	1.5869	0.0019		
24	Lamp1	P11438	Lysosome-associated membrane glycoprotein 1	− 1.6992	0.0005		
25	Dmd	P11531	Dystrophin			− 1.1485	0.0113
26	Itpr1	P11881	Inositol 1 4 5-trisphosphate receptor type 1	− 2.8966	0.0000	− 3.9266	0.0000
27	Scg5	P12961	Neuroendocrine protein 7B2	− 1.0625	0.0157	− 1.0625	0.0157
28	Calr	P14211	Calreticulin	− 1.2384	0.0064		
29	Atp6v0a2	P15920	V-type proton ATPase 116 kDa subunit a2	1.0728	0.0188		
30	Chgb	P16014	Secretogranin-1			− 1.8487	0.0004
31	Ap2a1	P17426	AP-2 complex subunit alpha-1			− 2.8336	0.0000
32	Ap2a2	P17427	AP-2 complex subunit alpha-2			− 2.2108	0.0001
33	Hexb	P20060	Beta-hexosaminidase subunit beta	− 1.5719	0.0011	− 1.5963	0.0008
34	Gria1	P23818	Glutamate receptor 1	− 1.4220	0.0034	− 3.0965	0.0000
35	Gria2	P23819	Glutamate receptor 2			− 1.8570	0.0004
36	Th	P24529	Tyrosine 3-monooxygenase	− 2.2049	0.0001	− 4.2800	0.0000
37	Pdia3	P27773	Protein disulfide-isomerase A3			− 1.2051	0.0073
38	Kif1a	P33173	Kinesin-like protein KIF1 A	2.6444	0.0000	1.9352	0.0007
39	Grin2a	P35436	Glutamate receptor ionotropic NMDA 2 A			− 1.3981	0.0038
40	Dnm1	P39053	Dynamin-1	− 2.9493	0.0000	− 3.8280	0.0000
41	Rph3a	P47708	Rabphilin-3 A	− 1.8070	0.0004		
42	Anxa5	P48036	Annexin A5			− 1.2122	0.0073
43	Cav1	P49817	Caveolin-1			− 1.3372	0.0047
44	Arsa	P50428	Arylsulfatase A	− 1.6005	0.0008	− 1.7866	0.0004
45	Atp6v1a	P50516	V-type proton ATPase catalytic subunit A	1.1758	0.0114		
46	Rab7a	P51150	Ras-related protein Rab-7a			− 1.4448	0.0026
47	Ptpn5	P54830	Tyrosine-protein phosphatase non-receptor type 5	− 1.2534	0.0060	− 1.5666	0.0012
48	Rab8a	P55258	Ras-related protein Rab-8 A			1.0916	0.0180
49	Rab4a	P56371	Ras-related protein Rab-4 A	1.0294	0.0221	1.1407	0.0146
50	Ncstn	P57716	Nicastrin	1.4921	0.0030		
51	Snap25	P60879	Synaptosomal-associated protein 25	1.2229	0.0090	− 2.0157	0.0002
52	Rab10	P61027	Ras-related protein Rab-10			1.4743	0.0034
53	Rab8b	P61028	Ras-related protein Rab-8B			1.4187	0.0040
54	Stx1b	P61264	Syntaxin-1B	1.5573	0.0023	1.0951	0.0179
55	Rab3a	P63011	Ras-related protein Rab-3 A	1.1857	0.0111		
56	Hspa8	P63017	Heat shock cognate 71 kDa protein	− 4.4586	0.0000	− 2.9272	0.0000
57	Vamp3	P63024	Vesicle-associated membrane protein 3	1.2269	0.0089		
58	Hspd1	P63038	60 kDa heat shock protein mitochondrial	− 4.7414	0.0000	− 2.4790	0.0000
59	Vamp2	P63044	Vesicle-associated membrane protein 2	2.0911	0.0004		
60	Cct6a	P80317	T-complex protein 1 subunit zeta	− 2.0976	0.0002	− 1.7460	0.0005
61	Ptprn2	P80560	Receptor-type tyrosine-protein phosphatase N2			1.2275	0.0090
62	Ap2 m1	P84091	AP-2 complex subunit mu			− 2.4742	0.0000
63	Anxa11	P97384	Annexin A11			− 1.7031	0.0005
64	Slc30a3	P97441	Zinc transporter 3			− 1.0511	0.0160
65	Apba2	P98084	Amyloid-beta A4 precursor protein-binding family A member 2			1.2206	0.0100
66	Grin2b	Q01097	Glutamate receptor ionotropic NMDA 2B	1.1603	0.0139		
67	Smpd1	Q04519	Sphingomyelin phosphodiesterase	− 1.5247	0.0018		
68	Anxa7	Q07076	Annexin A7	− 1.9531	0.0003	− 1.6067	0.0008
69	Unc13a	Q4 KUS2	Protein unc-13 homolog A			− 1.1920	0.0077
70	Rab26	Q504M8	Ras-related protein Rab-26			1.5954	0.0018
71	Lrrk2	Q5S006	Leucine-rich repeat serine/threonine-protein kinase 2	− 2.3484	0.0000	− 3.2565	0.0000
72	Akt2	Q60823	RAC-beta serine/threonine-protein kinase	1.4003	0.0043		
73	Vdac3	Q60931	Voltage-dependent anion-selective channel protein 3	− 1.0536	0.0160		
74	Snap91	Q61548	Clathrin coat assembly protein AP180	− 1.7470	0.0005	− 2.0284	0.0002
75	Dlg4	Q62108	Disks large homolog 4			− 1.3676	0.0041
76	Dpysl3	Q62188	Dihydropyrimidinase-related protein 3	− 1.0813	0.0143	1.2905	0.0073
77	Vamp1	Q62442	Vesicle-associated membrane protein 1	1.6719	0.0014		
78	Syn2	Q64332	Synapsin-2			− 1.8688	0.0004
79	Cbarp	Q66L44	Voltage-dependent calcium channel beta subunit-associated regulatory protein	1.6992	0.0013		
80	Sv2c	Q69ZS6	Synaptic vesicle glycoprotein 2 C	1.1222	0.0167	1.9166	0.0007
81	Sri	Q6P069	Sorcin			− 2.2502	0.0001
82	Akap7	Q7 TN79	A-kinase anchor protein 7 isoform gamma	1.2903	0.0073		
83	Actn1	Q7 TPR4	Alpha-actinin-1	− 4.5153	0.0000	− 5.8706	0.0000
84	Amph	Q7 TQF7	Amphiphysin			− 1.6787	0.0006
85	Map6	Q7 TSJ2	Microtubule-associated protein 6	2.7255	0.0000	1.8609	0.0007
86	Cadps	Q80 TJ1	Calcium-dependent secretion activator 1	− 1.1221	0.0131		
87	Sv2b	Q8BG39	Synaptic vesicle glycoprotein 2B			− 1.4133	0.0036
88	Slc6a17	Q8BJI1	Sodium-dependent neutral amino acid transporter SLC6 A17			− 1.2584	0.0058
89	Baiap2	Q8BKX1	Brain-specific angiogenesis inhibitor 1-associated protein 2			− 1.4786	0.0022
90	Dmxl2	Q8BPN8	DmX-like protein 2	− 1.2700	0.0057	− 3.0828	0.0000
91	Vps13c	Q8BX70	Vacuolar protein sorting-associated protein 13 C	− 2.4616	0.0000	− 1.6208	0.0008
92	Fndc3a	Q8BX90	Fibronectin type-III domain-containing protein 3 A			− 1.6146	0.0008
93	Ston2	Q8BZ60	Stonin-2			1.3964	0.0044
94	Vwf	Q8 CIZ8	von Willebrand factor	1.8817	0.0007	− 1.2443	0.0063
95	Scamp1	Q8 K021	Secretory carrier-associated membrane protein 1			− 1.2955	0.0054
96	Cyp51a1	Q8 K0 C4	Lanosterol 14-alpha demethylase			− 1.7877	0.0004
97	Fgb	Q8 K0E8	Fibrinogen beta chain			1.2247	0.0090
98	Unc13c	Q8 K0 T7	Protein unc-13 homolog C			− 1.2683	0.0057
99	Dnm1 l	Q8 K1M6	Dynamin-1-like protein	− 2.4497	0.0000	− 1.8255	0.0004
100	Rab15	Q8 K386	Ras-related protein Rab-15			1.6966	0.0013
101	Lgi3	Q8 K406	Leucine-rich repeat LGI family member 3			− 1.1139	0.0133
102	Snap47	Q8R570	Synaptosomal-associated protein 47			− 1.6228	0.0007
103	Fgg	Q8 VCM7	Fibrinogen gamma chain			3.1705	0.0000
104	Myh9	Q8 VDD5	Myosin-9			− 1.1162	0.0133
105	Dlg2	Q91XM9	Disks large homolog 2	− 1.1329	0.0123	− 1.5342	0.0016
106	Rab4b	Q91ZR1	Ras-related protein Rab-4B			1.2445	0.0087
107	Lrp1	Q91ZX7	Prolow-density lipoprotein receptor-related protein 1			− 2.3717	0.0000
108	Syt17	Q920M7	Synaptotagmin-17			− 2.0659	0.0002
109	Syt12	Q920 N7	Synaptotagmin-12			− 1.4071	0.0038
110	Myo5a	Q99104	Unconventional myosin-Va			− 2.7008	0.0000
111	Gars1	Q9 CZD3	Glycine–tRNA ligase	2.1563	0.0004		
112	Rab3b	Q9 CZT8	Ras-related protein Rab-3B	1.3236	0.0065	1.0544	0.0210
113	Tmed10	Q9D1D4	Transmembrane emp24 domain-containing protein 10	− 1.2406	0.0064	− 1.1264	0.0131
114	Gnai3	Q9DC51	Guanine nucleotide-binding protein G(i) subunit alpha-3	− 3.5140	0.0000	1.2626	0.0081
115	Rab13	Q9DD03	Ras-related protein Rab-13	1.6863	0.0014	1.5706	0.0022
116	Sv2a	Q9 JIS5	Synaptic vesicle glycoprotein 2 A	1.8292	0.0009		
117	Stx6	Q9 JKK1	Syntaxin-6			− 1.0870	0.0140
118	Rab37	Q9 JKM7	Ras-related protein Rab-37			1.3984	0.0044
119	Spag6	Q9 JLI7	Sperm-associated antigen 6			4.0086	0.0000
120	Tmed2	Q9R0Q3	Transmembrane emp24 domain-containing protein 2	1.4201	0.0040		
121	Atp6v1 g2	Q9 WTT4	V-type proton ATPase subunit G 2			− 1.1741	0.0079
122	Itpr2	Q9Z329	Inositol 1 4 5-trisphosphate receptor type 2	− 1.2895	0.0055	− 1.6527	0.0006

Abbreviations: *THRSP* thyroid hormone-responsive protein; *OE* overexpressed; *KO* knockout; *WT* wild-type; *DEPs* differentially expressed proteins; *STN* signal-to-noise ratio

### Gene Ontology (GO) Analysis

**Fig. 2:**
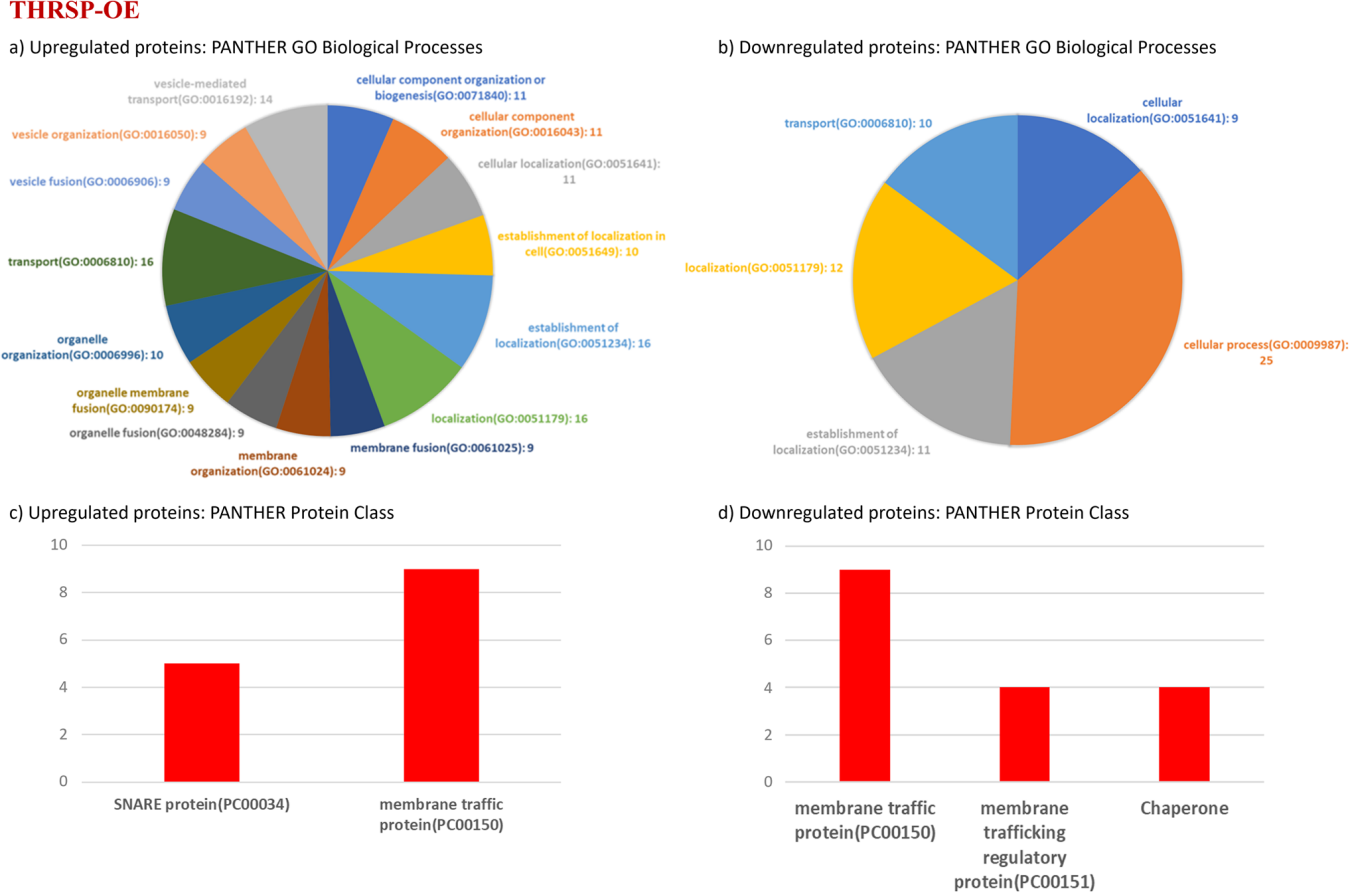
Molecular functions involved in the differentially expressed proteins in THRSP-OE relative to WT mice based on the Proteomics data (*n* = 6/group). **a**–**b** The GO biological processes identified from the upregulated and downregulated proteins of THRSP-OE mice by proteomics. The biological processes with the five highest number of involved proteins are shown here. **c**–**d** Protein classifications of the upregulated and downregulated proteins identified. All samples were uploaded and analyzed using the PANTHER GO classification system (v.18). Refer to Supplementary Tables 1 and 2 for the complete list of PANTHER GO biological processes identified from the upregulated and downregulated proteins in THRSP-OE mice

**Fig. 3:**
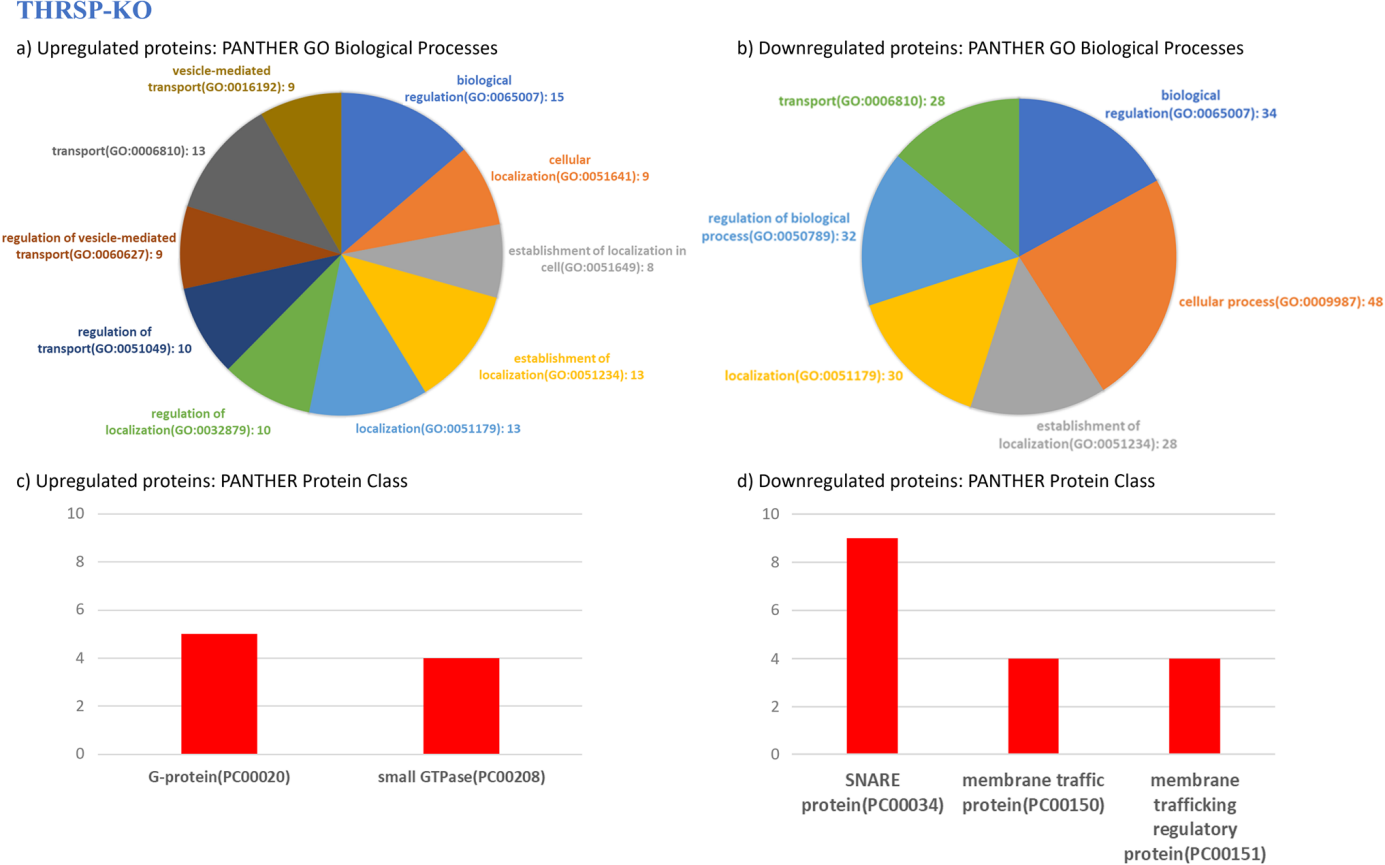
Molecular functions involved in the differentially expressed proteins in THRSP-KO relative to WT mice based on the Proteomics data (*n* = 6/group). **a**–**b** The GO biological processes identified from the upregulated and downregulated proteins of THRSP-OE mice by proteomics. The biological processes with the five highest number of involved proteins are shown here. **c**–**d** Protein classifications of the upregulated and downregulated proteins identified. All samples were uploaded and analyzed using the PANTHER GO classification system (v.18). Refer to Supplementary Tables 3 and 4 for the complete list of PANTHER GO biological processes identified from the upregulated and downregulated proteins in THRSP-KO mice

Analyses of GO biological processes (Figs. 2a, 2b, 3a, 3b), protein classifications (Figs. 2c, 2 d, 3c, 3 d), enriched pathways (Tables 2, 3), molecular functions (Table 4), and reactome pathways (Table 5) were performed using the PANTHER GO classification system (v.18) [12] using the identified DEPs from THRSP-OE and THRSP-KO mice relative to WT mice. Refer to Supplementary Tables 1, 2, 3, and 4 for the complete list of PANTHER GO biological processes identified from the upregulated and downregulated proteins in THRSP-OE and THRSP-KO mice.

**Table 2:** Enriched (PANTHER) pathways of all striatal DEPs identified in THRSP-OE and THRSP-KO mice relative to the WT mice

Enriched (PANTHER) Pathways	Mus musculus—reference list (21,983)	DEP	DEP (over/under)	DEP (fold enrichment)	DEP (FDR; *p*-value)
Synaptic vesicle trafficking (P05734)	29	11	+	67.24	0.0000
Adrenaline and noradrenaline biosynthesis (P00001)	32	10	+	55.4	0.0000
5HT3 type receptor mediated signaling pathway (P04375)	16	5	+	55.4	0.0000
Alpha adrenergic receptor signaling pathway (P00002)	23	7	+	53.96	0.0000
Plasminogen activating cascade (P00050)	16	3	+	33.24	0.0008
Ionotropic glutamate receptor pathway (P00037)	48	9	+	33.24	0.0000
Metabotropic glutamate receptor group III pathway (P00039)	68	12	+	31.29	0.0000
Metabotropic glutamate receptor group II pathway (P00040)	46	8	+	30.83	0.0000
Muscarinic acetylcholine receptors 1 and 3 signaling pathway (P00042)	59	10	+	30.05	0.0000
Opioid prodynorphin pathway (P05916)	36	6	+	29.55	0.0000
Metabotropic glutamate receptor group I pathway (P00041)	24	4	+	29.55	0.0001
Beta3 adrenergic receptor signaling pathway (P04379)	30	5	+	29.55	0.0000
Opioid proopiomelanocortin pathway (P05917)	37	6	+	28.75	0.0000
Opioid proenkephalin pathway (P05915)	37	6	+	28.75	0.0000
Dopamine receptor mediated signaling pathway (P05912)	57	9	+	27.99	0.0000
5HT1 type receptor mediated signaling pathway (P04373)	46	7	+	26.98	0.0000
Corticotropin releasing factor receptor signaling pathway (P04380)	34	5	+	26.07	0.0000
5HT4 type receptor mediated signaling pathway (P04376)	35	5	+	25.33	0.0000
Muscarinic acetylcholine receptors 2 and 4 signaling pathway (P00043)	58	8	+	24.45	0.0000
Beta2 adrenergic receptor signaling pathway (P04378)	46	6	+	23.12	0.0000
Beta1 adrenergic receptor signaling pathway (P04377)	46	6	+	23.12	0.0000
Nicotine pharmacodynamics pathway (P06587)	35	4	+	20.26	0.0004
Nicotinic acetylcholine receptor signaling pathway (P00044)	94	8	+	15.09	0.0000
Oxytocin receptor mediated signaling pathway (P04391)	60	5	+	14.77	0.0002
Thyrotropin-releasing hormone receptor signaling pathway (P04394)	63	5	+	14.07	0.0003
Heterotrimeric G-protein signaling pathway-rod outer segment phototransduction (P00028)	38	3	+	14	0.0072
Blood coagulation (P00011)	51	4	+	13.9	0.0013
5HT2 type receptor mediated signaling pathway (P04374)	68	5	+	13.04	0.0004
B cell activation (P00010)	71	5	+	12.48	0.0004
Endothelin signaling pathway (P00019)	82	4	+	8.65	0.0067
Huntington disease (P00029)	148	7	+	8.38	0.0002
T cell activation (P00053)	85	4	+	8.34	0.0071
Parkinson disease (P00049)	94	4	+	7.54	0.0099
CCKR signaling map (P06959)	163	6	+	6.53	0.0020
Inflammation mediated by chemokine and cytokine signaling pathway (P00031)	262	8	+	5.41	0.0008
Gonadotropin-releasing hormone receptor pathway (P06664)	235	7	+	5.28	0.0021
Unclassified (UNCLASSIFIED)	19,388	70	-	0.64	0.0000

Abbreviations: *THRSP* thyroid hormone-responsive protein; *OE* overexpressed; *KO* knockout; *WT* wild-type; *DEPs* differentially expressed proteins; *FDR* false discovery rate

**Table 3:** Specific proteins belonging to the enriched (PANTHER) pathways analyzed from all striatal DEPs identified in THRSP-OE and THRSP-KO mice relative to the WT mice

Enriched (PANTHER) Pathways	DEPs
Synaptic vesicle trafficking (P05734)	Syn2, Syn1, Stx1b, Unc13a, Syt12, Stxbp1, Snap25, Unc13c, Vamp1, Rab3a, Vamp2
Adrenaline and noradrenaline biosynthesis (P00001)	Th, Snap23, Snap25, Stx7, Slc6a17, Vamp1, Vti1b, Vamp3, Vamp2, Ddc
5HT3 type receptor mediated signaling pathway (P04375)	Snap23, Snap25, Vamp1, Vamp3, Vamp2
Alpha adrenergic receptor signaling pathway (P00002)	Stx6, Snap23, Snap25, Vamp1, Vamp3, Itpr1, Vamp2
Plasminogen activating cascade (P00050)	Fgb, Fgg, Fga
Ionotropic glutamate receptor pathway (P00037)	Grin2a, Gria1, Snap23, Grin2b, Snap25, Vamp1, Vamp3, Vamp2, Gria2
Metabotropic glutamate receptor group III pathway (P00039)	Grin2a, Prkaca, Gnai3, Gria1, Stx1b, Snap23, Grin2b, Snap25, Vamp1, Vamp3, Vamp2, Gria2
Metabotropic glutamate receptor group II pathway (P00040)	Prkaca, Gnai3, Stx1b, Snap23, Snap25, Vamp1, Vamp3, Vamp2
Muscarinic acetylcholine receptors 1 and 3 signaling pathway (P00042)	Grin2a, Itpr2, Stx1b, Snap23, Grin2b, Snap25, Vamp1, Vamp3, Itpr1, Vamp2
Opioid prodynorphin pathway (P05916)	Gnai3, Snap23, Snap25, Vamp1, Vamp3, Vamp2
Metabotropic glutamate receptor group I pathway (P00041)	Grin2a, Prkaca, Grin2b, Itpr1
Beta3 adrenergic receptor signaling pathway (P04379)	Snap23, Snap25, Vamp1, Vamp3, Vamp2
Opioid proopiomelanocortin pathway (P05917)	Gnai3, Snap23, Snap25, Vamp1, Vamp3, Vamp2
Opioid proenkephalin pathway (P05915)	Gnai3, Snap23, Snap25, Vamp1, Vamp3, Vamp2
Dopamine receptor mediated signaling pathway (P05912)	Th, Prkaca, Gnai3, Snap23, Snap25, Vamp1, Vamp3, Vamp2, Ddc
5HT1 type receptor mediated signaling pathway (P04373)	Prkaca, Gnai3, Snap23, Snap25, Vamp1, Vamp3, Vamp2
Corticotropin releasing factor receptor signaling pathway (P04380)	Snap23, Snap25, Vamp1, Vamp3, Vamp2
5HT4 type receptor mediated signaling pathway (P04376)	Snap23, Snap25, Vamp1, Vamp3, Vamp2
Muscarinic acetylcholine receptors 2 and 4 signaling pathway (P00043)	Prkaca, Gnai3, Stx1b, Snap23, Snap25, Vamp1, Vamp3, Vamp2
Beta2 adrenergic receptor signaling pathway (P04378)	Prkaca, Snap23, Snap25, Vamp1, Vamp3, Vamp2
Beta1 adrenergic receptor signaling pathway (P04377)	Prkaca, Snap23, Snap25, Vamp1, Vamp3, Vamp2
Nicotine pharmacodynamics pathway (P06587)	Th, Prkaca, Gnai3, Ddc
Nicotinic acetylcholine receptor signaling pathway (P00044)	Myo5a, Stx1b, Snap23, Snap25, Myh9, Vamp1, Vamp3, Vamp2
Oxytocin receptor mediated signaling pathway (P04391)	Snap23, Snap25, Vamp1, Vamp3, Vamp2
Thyrotropin-releasing hormone receptor signaling pathway (P04394)	Snap23, Snap25, Vamp1, Vamp3, Vamp2
Heterotrimeric G-protein signaling pathway-rod outer segment phototransduction (P00028)	Prkaca, Calm3, Calm1
Blood coagulation (P00011)	Fgb, Fgg, Fga, Vwf
5HT2 type receptor mediated signaling pathway (P04374)	Snap23, Snap25, Vamp1, Vamp3, Vamp2
B cell activation (P00010)	Itpr2, Atp6v1 g2, Calm3, Calm1, Itpr1
Endothelin signaling pathway (P00019)	Prkaca, Itpr2, Akt2, Itpr1
Huntington disease (P00029)	Grin2a, Grin2b, Dlg4, Rab8a, Akt2, Ap2a2, Ap2a1
T cell activation (P00053)	Akt2, Calm3, Calm1, Itpr1
Parkinson disease (P00049)	Snca, Th, Stx7, Hspa8
CCKR signaling map (P06959)	Dnm1, Prkaca, Ap2 m1, Calm1, Itpr1, Itgb1
Inflammation mediated by chemokine and cytokine signaling pathway (P00031)	Prkaca, Gnai3, Itpr2, Myh9, Akt2, Itpr1, Vwf, Itgb1
Gonadotropin-releasing hormone receptor pathway (P06664)	Dnm1, Gnai3, Itpr2, Anxa5, Cav1, Itpr1, Itgb1
Unclassified (UNCLASSIFIED)	Arsa, Snap47, Atp2b1, Cttnbp2, Vamp4, Dmxl2, Dmd, Lrrk2, Sri, Map6, Cadps, Ptprs, Scg5, Dlg2, Rab26, Kif1a, Rph3a, Atp6v0a2, Cyp51a1, Cbarp, Rab4b, Bin1, Lgi3, Rab7a, Dnm1 l, Rab15, Atp6v1a, Syt17, Rab13, Baiap2, Cct6a, Rab3b, Pdia3, Anxa11, Sparc, Vdac3, Kit, Hspd1, Chgb, Ptprn2, Ston2, Ctsl, Slc30a3, Spag6, Scamp1, Sv2b, Gars1, Amph, Akap7, Tmed10, Rab4a, Fndc3a, Hexb, Dennd4c, Tmed2, Ptpn5, Sv2c, Anxa7, Rab8b, Lamp1, Mctp1, Smpd1, Unc13, Snap91, Vps13c, Calr, Rab37, Sv2a, Spag6, Dgki

Abbreviations: *THRSP* thyroid hormone-responsive protein; *OE* overexpressed; *KO* knockout; *WT* wild-type; *DEPs* differentially expressed proteins

**Table 4:** PANTHER GO molecular functions of all striatal DEPs identified in THRSP-OE and THRSP-KO mice relative to the WT mice

PANTHER GO (Molecular Functions)	Mus musculus—reference list (21,983)	DEP	DEPs (over/under)	DEP (fold enrichment)	DEP (FDR; *p*-value)
syntaxin binding (GO:0019905)	30	11	+	65	0.0000
SNAP receptor activity (GO:0005484)	30	10	+	59.09	0.0000
SNARE binding (GO:0000149)	78	17	+	38.64	0.0000
protein kinase A regulatory subunit binding (GO:0034237)	12	2	+	29.55	0.0368
glutamate receptor activity (GO:0008066)	25	4	+	28.37	0.0006
collagen binding (GO:0005518)	13	2	+	27.27	0.0388
clathrin binding (GO:0030276)	47	6	+	22.63	0.0000
protein-macromolecule adaptor activity (GO:0030674)	111	13	+	20.76	0.0000
molecular adaptor activity (GO:0060090)	111	13	+	20.76	0.0000
calmodulin binding (GO:0005516)	43	3	+	12.37	0.0319
calcium ion binding (GO:0005509)	195	13	+	11.82	0.0000
GTPase activity (GO:0003924)	198	12	+	10.74	0.0000
ligand-gated channel activity (GO:0022834)	105	6	+	10.13	0.0011
ligand-gated monoatomic ion channel activity (GO:0015276)	105	6	+	10.13	0.0011
ribonucleoside triphosphate phosphatase activity (GO:0017111)	265	13	+	8.7	0.0000
metal ion binding (GO:0046872)	298	14	+	8.33	0.0000
neurotransmitter receptor activity (GO:0030594)	87	4	+	8.15	0.0279
pyrophosphatase activity (GO:0016462)	297	13	+	7.76	0.0000
hydrolase activity, acting on acid anhydrides, in phosphorus-containing anhydrides (GO:0016818)	298	13	+	7.73	0.0000
hydrolase activity, acting on acid anhydrides (GO:0016817)	298	13	+	7.73	0.0000
cation binding (GO:0043169)	331	14	+	7.5	0.0000
microtubule binding (GO:0008017)	148	6	+	7.19	0.0056
phospholipid binding (GO:0005543)	173	7	+	7.17	0.0019
tubulin binding (GO:0015631)	186	6	+	5.72	0.0141
ion binding (GO:0043167)	637	19	+	5.29	0.0000
monoatomic ion gated channel activity (GO:0022839)	235	7	+	5.28	0.0093
gated channel activity (GO:0022836)	235	7	+	5.28	0.0090
cytoskeletal protein binding (GO:0008092)	453	12	+	4.7	0.0004
lipid binding (GO:0008289)	280	7	+	4.43	0.0216
inorganic cation transmembrane transporter activity (GO:0022890)	368	9	+	4.34	0.0064
anion binding (GO:0043168)	333	7	+	3.73	0.0394
monoatomic ion channel activity (GO:0005216)	335	7	+	3.7	0.0375
monoatomic ion transmembrane transporter activity (GO:0015075)	496	10	+	3.57	0.0114
inorganic molecular entity transmembrane transporter activity (GO:0015318)	453	9	+	3.52	0.0210
protein binding (GO:0005515)	2397	44	+	3.25	0.0000
hydrolase activity (GO:0016787)	1411	20	+	2.51	0.0034
binding (GO:0005488)	6071	62	+	1.81	0.0000
molecular_function (GO:0003674)	12,130	86	+	1.26	0.0250
Unclassified (UNCLASSIFIED)	9853	38	-	0.68	0.0243
heterocyclic compound binding (GO:1,901,363)	2452	4	-	0.29	0.0337
organic cyclic compound binding (GO:0097159)	2500	4	-	0.28	0.0338
DNA-binding transcription factor activity, RNA polymerase II-specific (GO:0000981)	1134	0	-	< 0.01	0.0384
transcription cis-regulatory region binding (GO:0000976)	1114	0	-	< 0.01	0.0406
DNA-binding transcription factor activity (GO:0003700)	1193	0	-	< 0.01	0.0306
transcription regulatory region nucleic acid binding (GO:0001067)	1114	0	-	< 0.01	0.0397
sequence-specific double-stranded DNA binding (GO:1,990,837)	1136	0	-	< 0.01	0.0378
double-stranded DNA binding (GO:0003690)	1185	0	-	< 0.01	0.0310
transcription regulator activity (GO:0140110)	1403	0	-	< 0.01	0.0114
DNA binding (GO:0003677)	1432	0	-	< 0.01	0.0084
nucleic acid binding (GO:0003676)	2114	0	-	< 0.01	0.0003
sequence-specific DNA binding (GO:0043565)	1183	0	-	< 0.01	0.0318

Abbreviations: *GO* gene ontology; *THRSP* thyroid hormone-responsive protein; *OE* overexpressed; *KO* knockout; *WT* wild-type; *DEPs* differentially expressed proteins; *FDR* false discovery rate

**Table 5:** Reactome pathways analyzed from the differentially regulated and commonly upregulated/downregulated proteomics profile of THRSP transgenic (OE and KO) mice

Reactome Pathways	Mus musculus—reference list (21,983)	Analyzed list (37)	Fold enrichment	Raw *p*-value	FDR (*p*-value)
Unclassified (UNCLASSIFIED)	12,918	8	0.37	0.0000	0.0025
Hemostasis (R-MMU-109582)	624	7	6.66	0.0001	0.0129
Vesicle-mediated transport (R-MMU-5653656)	708	10	8.39	0.0000	0.0002
Neuronal System (R-MMU-112316)	338	6	10.55	0.0000	0.0074
Membrane Trafficking (R-MMU-199991)	561	10	10.59	0.0000	0.0000
Platelet activation, signaling and aggregation (R-MMU-76002)	253	5	11.74	0.0001	0.0131
Neurotransmitter receptors and postsynaptic signal transmission (R-MMU-112314)	146	4	16.28	0.0001	0.0152
Transmission across Chemical Synapses (R-MMU-112315)	212	6	16.82	0.0000	0.0009
Clathrin-mediated endocytosis (R-MMU-8856828)	140	4	16.98	0.0001	0.0141
Integration of energy metabolism (R-MMU-163685)	79	3	22.56	0.0004	0.0358
Regulation of insulin secretion (R-MMU-422356)	62	3	28.75	0.0002	0.0204
RAB geranylgeranylation (R-MMU-8873719)	62	3	28.75	0.0002	0.0219
Ion homeostasis (R-MMU-5578775)	50	3	35.65	0.0001	0.0151
Activation of NMDA receptors and postsynaptic events (R-MMU-442755)	37	3	48.17	0.0000	0.0089
Glucagon-like Peptide-1 (GLP1) regulates insulin secretion (R-MMU-381676)	32	3	55.7	0.0000	0.0079
Metabolism of amine-derived hormones (R-MMU-209776)	18	2	66.02	0.0005	0.0491
Elevation of cytosolic Ca2 + levels (R-MMU-139853)	13	2	91.41	0.0003	0.0307
Catecholamine biosynthesis (R-MMU-209905)	4	2	> 100	0.0000	0.0101

Abbreviations: *THRSP* thyroid hormone-responsive protein; *OE* overexpressed; *KO* knockout; *FDR* false discovery rate

### Protein–Protein Interactions Analysis via Search Tool for the Retrieval of Interacting Genes/Proteins (STRING)

**Fig. 4:**
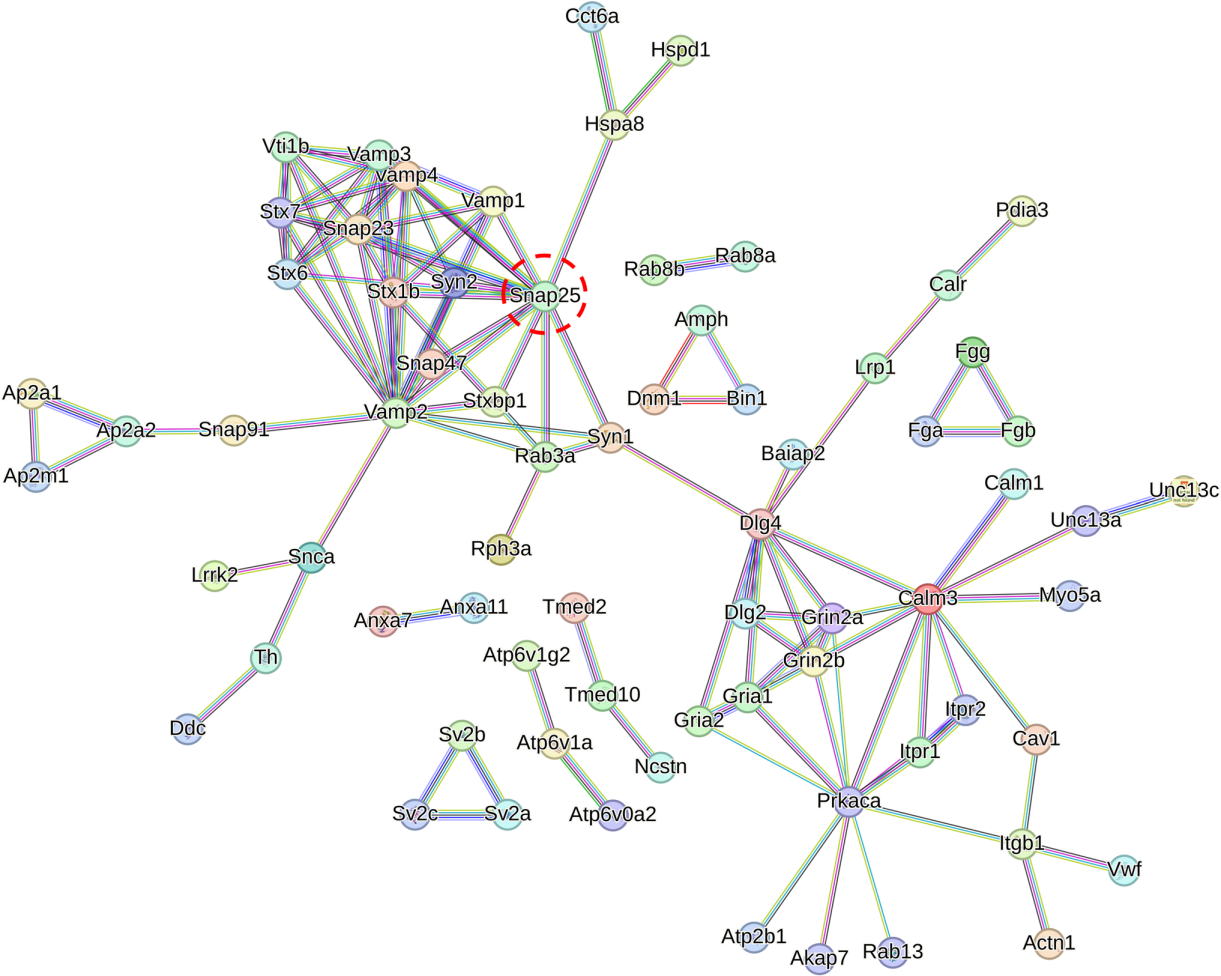
Protein–protein interaction of all identified DEPs in THRSP-OE and THRSP-KO mice relative to WT mice based on the Proteomics data (*n* = 6/group), analyzed by STRING. Only high-confidence interactions with the highest threshold score of 0.90 are shown. The nodes in the network that were disconnected are excluded

To understand the biological importance of the DEPs, protein–protein interaction networks were created using the STRING tool (v.12.0) [13]. A similar process was conducted based on our previous publication [7], which assessed protein–protein interaction using the same software. The protein networks (Fig. 4) generated were for all the DEPs identified in both the THRSP-OE and THRSP-KO models compared to the WT mice. Only high-confidence interactions, with the highest score of 0.90, were shown. The nodes in the network that were disconnected were excluded.

### RNA Extraction and RT-qPCR

**Fig. 5:**
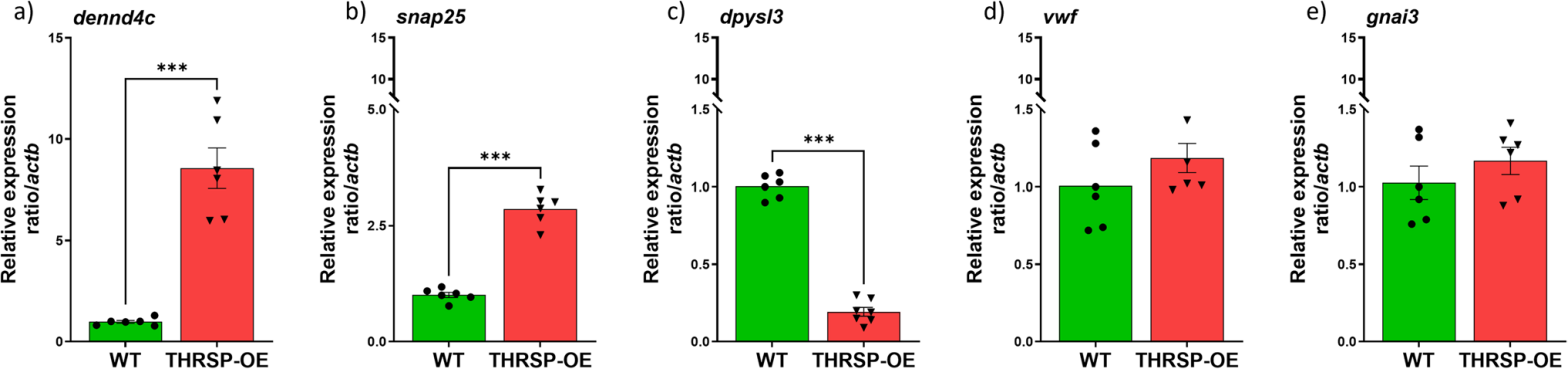
The mRNA expression levels of differentially regulated proteins identified in THRSP-OE mice. RT-qPCR of striatal tissues (*n* = 6 mice/group) was employed to measure mRNA expression levels of **a** *dennd4c* (unpaired *t*-test, *t* = 7.803, *df* = 5, *p* < 0.001); **b** *snap25* (unpaired *t*-test, *t* = 12.93, *df* = 5, *p* < 0.001); **c** *dpysl3* (unpaired *t*-test, *t* = 35.09, *df* = 5, *p* < 0.001); **d** *vwf* (unpaired *t*-test, *t* = 1.065, *df* = 3, *p* = 0.336); **e** *gnai3* (unpaired *t*-test, *t* = 0.7545, *df* = 5, *p* = 0.485). Values are presented as the mean ± standard error of the mean (SEM). ***p < 0.001, by *t*-test relative to WT mice. The genes analyzed here are based on Table 6

**Fig. 6:**
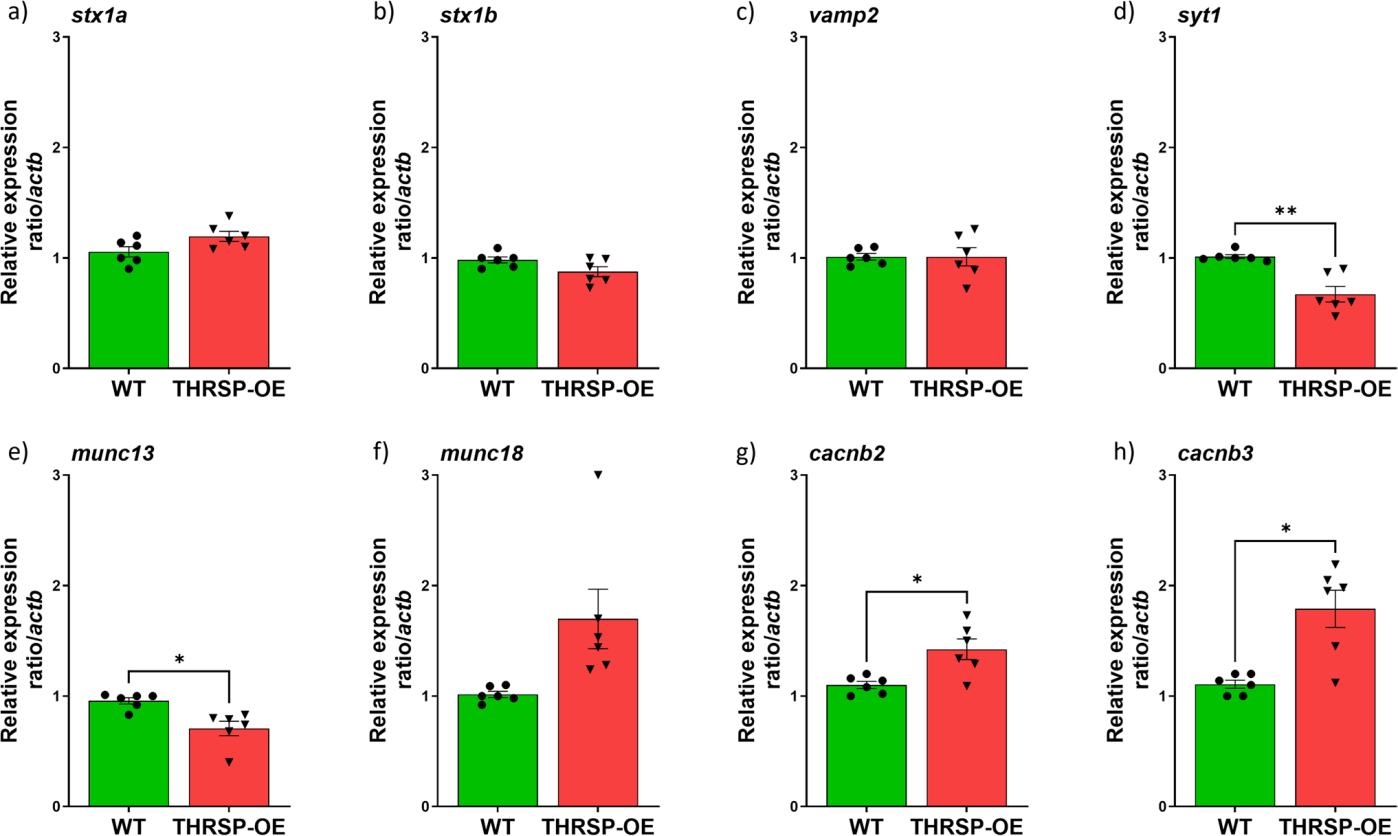
The mRNA expression levels of *snap25*-related genes in THRSP-OE mice primarily involved in SNARE protein complex. RT-qPCR of striatal tissues (*n* = 6 mice/group) was employed to measure mRNA expression levels of **a** *stx1a* (unpaired *t*-test, *t* = 1.962, *df* = 5, *p* = 0.107); **b** *stx1b* (unpaired *t*-test, *t* = 1.656, *df* = 5, *p* = 0.159); **c** *vamp2* (unpaired *t*-test, *t* = 3.832e-016, *df* = 5, *p* > 0.999); **d** *syt1* (unpaired *t*-test, *t* = 5.201, *df* = 5, *p* = 0.003); **e** *munc13* (unpaired *t*-test, *t* = 3.079, *df* = 5, *p* = 0.028); **f** *munc18* (unpaired *t*-test, *t* = 2.448, *df* = 5, *p* = 0.058); **g** *cacnb2* (unpaired *t*-test, *t* = 3.655, *df* = 5, *p* = 0.015); **h** *cacnb3* (unpaired *t*-test, *t* = 3.842, *df* = 5, *p* = 0.012). Values are presented as the mean ± standard error of the mean (SEM). *p < 0.05 and **p < 0.01 by *t*-test relative to WT mice

Based on our previous protocol [6], total RNA from striatal tissue samples from THRSP-OE and wild-type mice (*n* = 6/group) were isolated using TRIzol™ Reagent (Invitrogen, Carlsbad, CA, USA). A Hybrid-RTM Kit (Geneall Biotechnology, Seoul, Korea) was used for RNA purification. A Colibri Microvolume Spectrometer determined the total RNA concentration (Titertek-Berthold, Pforzheim, Germany). The RT-qPCR was employed to measure mRNA expression levels of differentially regulated proteins (Fig. 5, Table 6) and the corresponding SNARE protein complex-related targets (Fig. 6). A 2.5 µg of total RNA was reverse transcribed into cDNA using AccuPower CycleScript RT Premix (Bioneer, Seoul, Korea) according to the manufacturer’s instructions and performed on a BioRad T100 Thermal Cycler. The cDNA amplification was performed using custom-made sequence-specific primers (Cosmogenetech, Seoul, Korea) and was detected with SYBR Green (Solgent, Korea). RT-qPCR analysis was performed in duplicate using the Applied Biosystems Step-One Plus Real-Time PCR System, with optimized cycling conditions that included a denaturing step at 94 °C for 1 min, followed by annealing at a primer-specific temperature for 1 min, and elongation at 72 °C for 45 s, for a total of 45 cycles. The resulting values were then normalized to the expression levels of Actin beta (actb), a housekeeping gene commonly used as a reference for RT-qPCR analysis. The 2^−ΔΔCt^ formula was utilized to calculate the relative expression of target genes. A complete list of primers used in this study can be found in Supplementary Table 5.

**Table 6:** The differentially regulated and commonly upregulated and downregulated proteins in THRSP transgenic (OE and KO) mice based proteomic analysis

**Differentially regulated**						
Gene name	Accession	Description	THRSP-OE		THRSP-KO	
			STN ratio	*p*-value	STN ratio	*p*-value
Dennd4c	A6H8H2	DENN domain-containing protein 4 C	1.3632	0.0058	− 1.1291	0.0128
Snap25	P60879	Synaptosomal-associated protein 25	1.2229	0.0090	− 2.0157	0.0002
Dpysl3	Q62188	Dihydropyrimidinase-related protein 3	− 1.0813	0.0143	1.2905	0.0073
Vwf	Q8 CIZ8	von Willebrand factor	1.8817	0.0007	− 1.2443	0.0063
Gnai3	Q9DC51	Guanine nucleotide-binding protein G(i) subunit alpha-3	− 3.5140	0.0000	1.2626	0.0081
**Common upregulated**						
Gene name	Accession	Description	THRSP-OE		THRSP-KO	
			STN ratio	*p*-value	STN ratio	*p*-value
Kif1a	P33173	Kinesin-like protein KIF1 A	2.6444	0.0000	1.9352	0.0007
Rab4a	P56371	Ras-related protein Rab-4 A	1.0294	0.0221	1.1407	0.0146
Stx1b	P61264	Syntaxin-1B	1.5573	0.0023	1.0951	0.0179
Sv2c	Q69ZS6	Synaptic vesicle glycoprotein 2 C	1.1222	0.0167	1.9166	0.0007
Map6	Q7 TSJ2	Microtubule-associated protein 6	2.7255	0.0000	1.8609	0.0007
Rab3b	Q9 CZT8	Ras-related protein Rab-3B	1.3236	0.0065	1.0544	0.0210
Rab13	Q9DD03	Ras-related protein Rab-13	1.6863	0.0014	1.5706	0.0022
**Common downregulated**						
Gene name	Accession	Description	THRSP-OE		THRSP-KO	
			STN ratio	*p*-value	STN ratio	*p*-value
Bin1	O08539	Myc box-dependent-interacting protein 1	− 1.8097	0.0004	− 3.2773	0.0000
Ddc	O88533	Aromatic-L-amino-acid decarboxylase	− 3.5795	0.0000	− 3.3286	0.0000
Syn1	O88935	Synapsin-1	− 1.5979	0.0008	− 2.3051	0.0001
Prkaca	P05132	cAMP-dependent protein kinase catalytic subunit alpha	− 2.5336	0.0000	− 1.5504	0.0013
Itpr1	P11881	Inositol 1 4 5-trisphosphate receptor type 1	− 2.8966	0.0000	− 3.9266	0.0000
Scg5	P12961	Neuroendocrine protein 7B2	− 1.0625	0.0157	− 1.0625	0.0157
Hexb	P20060	Beta-hexosaminidase subunit beta	− 1.5719	0.0011	− 1.5963	0.0008
Gria1	P23818	Glutamate receptor 1	− 1.4220	0.0034	− 3.0965	0.0000
Th	P24529	Tyrosine 3-monooxygenase	− 2.2049	0.0001	− 4.2800	0.0000
Dnm1	P39053	Dynamin-1	− 2.9493	0.0000	− 3.8280	0.0000
Arsa	P50428	Arylsulfatase A	− 1.6005	0.0008	− 1.7866	0.0004
Ptpn5	P54830	Tyrosine-protein phosphatase non-receptor type 5	− 1.2534	0.0060	− 1.5666	0.0012
Hspa8	P63017	Heat shock cognate 71 kDa protein	− 4.4586	0.0000	− 2.9272	0.0000
Hspd1	P63038	60 kDa heat shock protein mitochondrial	− 4.7414	0.0000	− 2.4790	0.0000
Cct6a	P80317	T-complex protein 1 subunit zeta	− 2.0976	0.0002	− 1.7460	0.0005
Anxa7	Q07076	Annexin A7	− 1.9531	0.0003	− 1.6067	0.0008
Lrrk2	Q5S006	Leucine-rich repeat serine/threonine-protein kinase 2	− 2.3484	0.0000	− 3.2565	0.0000
Snap91	Q61548	Clathrin coat assembly protein AP180	− 1.7470	0.0005	− 2.0284	0.0002
Actn1	Q7 TPR4	Alpha-actinin-1	− 4.5153	0.0000	− 5.8706	0.0000
Dmxl2	Q8BPN8	DmX-like protein 2	− 1.2700	0.0057	− 3.0828	0.0000
Vps13c	Q8BX70	Vacuolar protein sorting-associated protein 13 C	− 2.4616	0.0000	− 1.6208	0.0008
Dnm1 l	Q8 K1M6	Dynamin-1-like protein	− 2.4497	0.0000	− 1.8255	0.0004
Dlg2	Q91XM9	Disks large homolog 2	− 1.1329	0.0123	− 1.5342	0.0016
Tmed10	Q9D1D4	Transmembrane emp24 domain-containing protein 10	− 1.2406	0.0064	− 1.1264	0.0131
Itpr2	Q9Z329	Inositol 1 4 5-trisphosphate receptor type 2	− 1.2895	0.0055	− 1.6527	0.0006

Abbreviations: *THRSP* thyroid hormone-responsive protein; *OE* overexpressed; *KO* knockout; *DEPs* differentially expressed proteins; *STN* signal-to-noise ratio

### Receptor Binding Affinity Studies

The striatal samples (for D1 and D2/D3 ligand binding) from THRSP-OE and wild-type mice (*n* = 5/group) were homogenized in a 20 × volume of buffer A (50 mM Tris–HCl, pH 7.4 at 4 C, 1 mM EDTA, 5 mM KCl, 1.5 mM Ca Cl2, 4 mM MgCl2, 120 mM NaCl) and centrifuged at 34,500 g for 10 min. The pellet (samples) was re-suspended in an equal volume of buffer A and re-centrifuged for 10 min at 34,500 g. The samples were then re-suspended in buffer A, at a concentration of 10 mg (wet tissue weight/mL) for immediate use. Subsequently, ligand binding for D1 receptors was performed with 2 nM concentrations of [3H] SCH23390 performed for 1H at room temperature (21 °C). Moreover, ligand binding for D2/D3 receptors was performed with 3 nM concentrations of [3H]-Spiperone for 2.5H at 4 °C based on our previous protocols with modifications [14, 15]. Afterwards, pellets were washed three times with ice-cold serum-free media, dissolved in 1% SDS, mixed with a liquid scintillation cocktail, and measured with a Wallac 1450 MicroBeta® TriLux liquid scintillation counter (PerkinElmer, Waltham, MA, USA) to determine the [3H] content for each sample. 

### Enzyme-Linked Immunosorbent Assay

To evaluate the total striatal tissue dopamine levels in mice (*n* = 8/group), we performed Dopamine ELISA (KA1887, Abnova, Taiwan), following our previously established protocol [16]. In brief, we prepared standards, controls, and samples using an extraction and acylation technique. A standard curve was generated using dopamine standards of known concentrations to quantify dopamine levels in the samples. After extraction, we added enzyme solution, supernatant (i.e., extracted standards, controls, and samples), and HCl into a 96-well dopamine microtiter plate, which was incubated at room temperature for 30 min. We then added dopamine anti-serum and incubated the plate at room temperature for 2 h. Following incubation, we discarded the samples and washed the plate three times with a wash buffer. Next, we incubated the plate with enzyme conjugate for 30 min, discarded it, and washed it three times. We then added TMB substrate and incubated the plate for 25 min, keeping it protected from direct light. Finally, we added the stop solution and measured absorbance at 450 nm using the EMax Plus Microplate Reader (Molecular Devices, San Jose, CA, USA). Dopamine concentrations in the samples were calculated by comparing the absorbance values to the standard curve generated from the known dopamine standards. We performed all measurements in duplicate to ensure accuracy.

### EEG Data Acquisition and Experimental Procedure

EEG was conducted to evaluate theta and beta waves in mice according to our previous study [8]. The mice were anesthetized using 0.02 mL of Zoletil® (50 mg/mL) and Rompun® (xylazine 23.32 mg/mL), and a three-channel tethered head mount (8200 K3-iS/iSE) was implanted. The head mount was fixed with two stainless screws in the frontal region (A/P − 1.0 mm, M/L − 1.5 mm) and two stainless screws in the posterior brain region (A/P − 1.0 mm, M/L ± 1.5 mm) using dental cement. We followed standard operating procedures to minimize animal suffering. After the implantation, the mice were given a 7-day recovery period before EEG recording. The mice were allowed to acclimatize to the EEG apparatus for 2 h (unrecorded) while attached to theter. The following day, we injected THRSP-OE and WT mice with a single administration of 5 mg/kg of methylphenidate (MPH), a first-line treatment for ADHD, and vehicle (VEH) (*n* = 6/group). Moreover, separate groups were injected with the same drug at varying doses (2, 5, 10 mg/kg) for 7 days to assess the effects of repeated MPH and VEH administration in the EEG in mice. The signals were adjusted to 400 Hz using a 14-bit A/D converter channeled to a computer-based software package, Sirenia® software suite (Pinnacle Technology, Inc., Lawrence, KS, USA).

The EEG data analyses were conducted on channel 2 A-B (after removing EMG and 1 A-B channels) using MATLAB (R2021b, MathWorks Inc.). The 1 A-B channel was removed due to signal attenuation that occurred in most mice data. The data were bandpass-filtered using a finite impulse response filter with a high-pass filter with a cutoff frequency of 1 Hz and a low-pass filter with a cutoff frequency of 30 Hz to isolate the frequencies of interest while eliminating environmental and muscular artifacts. The resulting data was then downsampled to 100 Hz. Eye blinks in the data were mitigated by filtering the identified eye blink intervals through a hard-thresholding discrete wavelet transform (DWT) and restoring the filtered signal via inverse DWT. For the spectral approaches, we computed the power spectral density (PSD) at different EEG frequencies using the MATLAB pwelch function (with a 10-s window, 1024 number of discrete Fourier transform points, and 50% overlap). Following PSD calculation, power within the delta (1–4 Hz), theta (5–8 Hz), alpha (9–12 Hz), and beta (13–30 Hz) frequency bands was quantified using logarithmic transformations to express the data in decibels. Finally, the TBR was computed, which could serve as a biomarker of cognitive state and neurological condition. The TBR is a reliable measure of ADHD, diagnosed based on the presence of a higher TBR in individuals with ADHD compared to those without the disorder [17]. This ratio is calculated by dividing the theta wave power by the beta wave power and is used as an objective indicator of ADHD symptoms. To assess the drug efficacy (methylphenidate 5 mg/kg) in EEG, we extracted data over a 40-min time window after 5 min of drug administration.

### Statistical Analysis

Statistical analyses were performed using GraphPad Prism v10.4.0 (GraphPad Software, Inc., La Jolla, CA, USA). For graphical purposes, data are presented as mean ± standard error of the mean, and all statistical analyses were conducted on raw data. The results were analyzed using either *t*-test or one-way analysis of variance (ANOVA), followed by Tukey’s multiple comparison test. A level of probability of *P* ≤ 0.05 was defined as the threshold for statistical significance. Experiments were replicated at least three times.

## Results

### Striatal Proteomic Analysis of Early Adult THRSP-OE Mouse Model of ADHD-PI Presentation

ADHD is a complex disorder that involves the interaction of many different genes and proteins, resulting in specific behavioral characteristics. Studies have shown that manipulating a single gene or protein can impact other genes and proteins, leading to symptoms such as inattention, hyperactivity, and impulsivity. Our previous study suggests that the predominantly inattentive THRSP-OE mice have intrinsic impairments in the Wnt signaling pathway, as reported through hippocampal proteomics [7], which predisposes this animal model to inattention.

To extend our findings, we conducted a proteomic study using the striatum in mice. The striatum is an important brain region for attention and is affected in individuals with ADHD [18]. It regulates the release of dopamine, a neurotransmitter that plays a vital role in attention, motivation, and reward. Interestingly, people with ADHD have lower levels of dopamine in the striatum, a dysfunction called “hypo-dopaminergic trait,” which may contribute to their attentional deficits [19]. Moreover, the striatum is connected to other brain regions involved in attentional processes, such as the prefrontal cortex [20]. Indeed, dysfunctions in the striatum and its connections to other brain regions can also contribute to attentional deficits in people with ADHD.

In the present study, a total of 122 DEPs were identified in THRSP-OE and THRSP-KO relative to WT mice (Fig. 1b; Table 1) and showing 37 common DEPs. Out of these, 32 and 35 proteins were upregulated and downregulated in THRSP-OE, respectively, whereas 23 and 69 proteins were upregulated and downregulated in THRSP-KO, respectively. These findings suggest that the overexpression or knockout of the THRSP gene can have a significant intrinsic impact on the expression of other proteins in mice. Using this information, we applied the PANTHER classification system to identify the GO biological processes involved in the DEPs. GO analyses of upregulated (Fig. 2a) and downregulated (Fig. 2b) proteins in THRSP-OE mice identified variations in vesicle-mediated transport, cellular localization, cellular processes, and transport, among others. On the other hand, the GO analyses of upregulated (Fig. 3a) and downregulated (Fig. 3b) proteins in THRSP-KO mice identified involvement in the regulation of vesicle-mediated transport, localization, and biological processes, among others.

These cellular and biological processes show the possibility of impaired synaptic signaling involvement, particularly with THRSP-OE mice, which could be related to the established predominant inattention observed in this ADHD mouse model [9]. However, it must be examined whether synaptic signaling is impaired in response to its transgenic nature. Therefore, using the DEPs both in the THRSP-OE and THRSP-KO mice, we conducted enrichment analysis using PANTHER pathways and identified enriched pathways, including synaptic vesicle trafficking and receptor-mediated signaling pathways (Table 2), including dopamine receptor-mediated signaling pathways known to be involved in ADHD [21]. Moreover, it is interesting to observe that the thyrotropin-releasing hormone receptor signaling pathway was already identified, which further supports the role of thyroid hormone signaling in ADHD [22, 23], which we previously evaluated in the same mouse model [8].

Using the enriched pathways identified, we have further scrutinized what proteins were involved in the presented cellular and biological pathways, and we were intrigued to find out that most recurring sets of proteins involved were those with contributory roles in plasma membrane and synaptic vesicle regulation (Table 3), particularly the Snap25 protein, a member of the family of proteins that make up the soluble N-ethylmaleimide-sensitive factor attachment protein receptors (SNARE) complex, known to have direct links to neurotransmitter regulation including dopamine [24] and previously identified to be involved in ADHD pathology both in humans [2, 25, 26] and animal models [27–29].

It has been shown that the DEPs in both the THRSP-OE and THRSP-KO mice belong to the SNARE proteins, member traffic proteins, and G-proteins classification (as shown in Figs. 2c, 2 d, 3c, and 3 d). This indicates that the changes in the THRSP gene, especially in THRSP-OE mice, have intrinsic effects on different molecular functions and pathways, particularly transduction and signaling (as outlined in Tables 4 and 5), among others, which may be responsible for the predominant inattention in this ADHD model.

Furthermore, the total DEPs were also analyzed using STRING, showing protein–protein interaction networks restricted to high-confidence (0.9) interaction thresholds only. The protein networks from THRSP-OE and THRSP-KO mice revealed the high interaction of Snap25 (Fig. 4) among other SNARE complex-related proteins. These critical findings provide evidence of differences between the two transgenic mice, particularly how Snap25 is among the five differentially regulated proteins (Table 5) in THRSP-OE and THRSP-KO mice to which the THRSP-OE mice show upregulation of Snap25. In contrast, THRSP-KO mice show downregulation of this protein. Of the DEPs identified in the study, the Snap25 protein is of particular interest given its important function in the SNARE protein complex by maintaining the complex’s assembly and stability and proper synaptic transmission and brain function, if impaired, can lead to impaired neurotransmitter release (including dopamine) and eventually inducing neuropsychiatric disorders [30], such as ADHD. Overall, the current focus is on understanding how the upregulation of Snap25 affects the regulation of the SNARE protein complex and its targets and evaluating its contributory effects on the established ADHD-PI-like behaviors observed in THRSP OE mice.

### The Differentially Regulated Proteins in THRSP OE Mice

Notably, five proteins were identified to be differentially regulated (Table 6) among all DEPs, meaning that these protein levels were opposing between the two mouse groups (THRSP-OE vs THRSP-KO). For instance, we observed that Dennd4c, Snap25, and Vwf proteins were upregulated in THRSP-OE mice, while these were downregulated in THRSP-KO mice. Conversely, Dpysl3 and Gnai3 proteins were downregulated in THRSP-OE and upregulated in THRSP-KO mice. Given these findings, we analyzed the differentially regulated proteins identified in striatal proteomics with a particular focus on the THRSP-OE mice, given that this animal model has an established ADHD-PI-like behavior and is the target of the study. Therefore, at this stage, we have conducted the mRNA analysis via RT-qPCR in *dennd4c*, *snap25*, *dpysl3*, *vwf*, and *gnai3* genes only in the ADHD-PI model, THRSP-OE mice.

It is worth noting that all five differentially regulated proteins play crucial roles in nervous system function, including neurotransmission, neuronal connectivity, and blood flow regulation. Dendd4c, also known as Ras-related protein Rab-10 (Rab10), is a protein that regulates endocytosis [31], a process that helps control the number of receptors on the surface of cells, including neurons and also found to regulate dendritic branching by balancing dendritic transport [32]. Snap25 is a SNARE protein family member that plays an essential role in neurotransmitter release at the synapse [5], a process necessary for communication between neurons. Dpysl3, also known as collapsin response mediator protein 4 (Crmp4), is involved in axon guidance [33], which helps ensure proper neuronal connectivity during development. Vwf, or von Willebrand factor, is involved in blood clotting, found to promote blood–brain barrier flexibility [34]. Finally, Gnai3 is a G protein subunit (inhibitory alpha subunit) that is a component of the G protein complex (alpha, beta, and gamma subunits), which plays a crucial role in signaling pathways regulating neurotransmitter release and synaptic plasticity. As a key regulator of G protein-coupled receptor signaling, Gnai3 has been implicated in the modulation of neuronal excitability and synaptic transmission, and its dysregulation has been linked to various neurological disorders [35].

Results show increased levels of *dennd4c* and *snap25* genes in the striatum of THRSP-OE mice compared to the WT mice (Fig. 5a, b). Moreover, there was a decreased expression of the *dpysl3* gene (Fig. 5c) in the striatum of THRSP-OE mice compared to the WT mice. This shows similar trends with the expression levels of the Dendd4c, Snap25, and Dpysl3 protein levels identified in the striatal proteomics. However, while we found similarities in the expression levels of protein and genes for the three mentioned above, we failed to see significant changes in the expression levels of the *vwf* and *gnai3* genes (Fig. 5d, e) in the striatum of THRSP-OE mice relative to the WT mice. Nonetheless, we found sustained expression levels of the *snap25* gene which is among the most important findings in this study, given its previous involvement in ADHD pathology [4, 26, 27, 29].

### The SNARE Complex

Our study has uncovered novel and sustained changes in the *snap25* gene expression levels in THRSP-OE mice. This discovery prompted us to delve deeper and examine the responses of the other genes involved in the SNARE complex and voltage-dependent Ca2 + channels (VDCC). The SNARE complex and VDCC, both of which are crucial in neurotransmitter release, are formed by the interaction of several proteins, including Vamp2 (located on the vesicle membrane), Snap25, and Stx1a and Stx1b (located on the presynaptic membrane) [36]. Functionally, this complex is responsible for the docking of synaptic vesicles at the presynaptic membrane and the subsequent fusion of the vesicle membrane with the presynaptic membrane through the recruitment of necessary proteins, such as Syt1, Munc13, and Munc18, leading to neurotransmitter release into the synaptic cleft. VDCC, on the other hand, allows Ca2 + influx into the presynaptic terminal, which triggers the release of neurotransmitters by the SNARE complex.

Given the innate upregulation of the Snap25 protein identified in the proteomics analysis and the resultant *snap25* gene overexpression by RT-qPCR, one would assume that the SNARE complex, where this gene or protein is highly involved, may be altered. However, it is interesting that the key players in the SNARE complex, namely the *stx1a*, *stx1b*, and *vamp2* genes, were not significantly altered (Fig. 6a, b, c). However, we only found a slightly increased expression level of *the stx1a* gene but a slightly reduced *stx1b* gene expression level relative to the wild-type. Moreover, we found no slight changes in the expression level of *the vamp2* gene. This is a quite intriguing result given that both the Stx1b and Vamp2 proteins were among the DEPs identified in the proteomics in which both the Stx1b (STN ratio = 1.5573; *p* = 0.0023) and Vamp2 (STN ratio = 2.0911; *p* = 0.0004) proteins were upregulated relative to the wild-type (refer to Table 1). The observed discrepancy between the protein and gene expression data, particularly between Stx1b protein and *stx1b* gene and Vamp2 protein and *vamp2* gene, suggests that post-transcriptional or post-translational modifications may be involved in regulating the expression or function of these proteins. Also, we observed significant reductions in *syt1* and *munc13* genes, but the *munc18* gene slightly increased, though it did not reach a significance level (Fig. 6d, e, f). The significant reductions in *syt1* and *munc13* gene expression suggest that changes in *snap25* expression may have downstream effects on other genes involved in the SNARE complex. These reductions in *syt1* and *munc13* gene expression could potentially lead to changes in the SNARE complex’s activity and the neurotransmitter release process. However, it is important to note that the downstream effects of changes in *snap25* gene expression on the function of the SNARE complex and neurotransmitter release are complex and may involve multiple genes and pathways.

Furthermore, two VDCC-related genes, namely *cacnb2* and *cacnb3* genes, were overexpressed in THRSP-OE mice (Fig. 6g, h). The exact mechanism by which Snap25 regulates the expression levels of *cacnb2* and *cacnb3* genes has yet to be fully understood. However, it is thought to be since Snap25 plays a critical role in regulating VDCC and calcium-dependent neurotransmitter release. The increase in the expression levels of *cacnb2* and *cacnb3* genes, when *snap25* is overexpressed, may be a compensatory mechanism to enhance the activity of the channels and promote efficient neurotransmitter release [37].

These findings underscore the complexities of the regulatory mechanisms involved in SNARE complex formation and function and the need for further studies to fully understand the molecular mechanisms underlying the observed effects of *snap25* gene overexpression in THRSP-OE mice on the SNARE complex and neurotransmitter release, particularly the dopamine. The unexpected results and the potential involvement of post-transcriptional or post-translational modifications in regulating the expression or function of these proteins further emphasize the intricacy of these processes.

### Dopamine Neurotransmission in THRSP-OE Mice

**Fig. 7:**
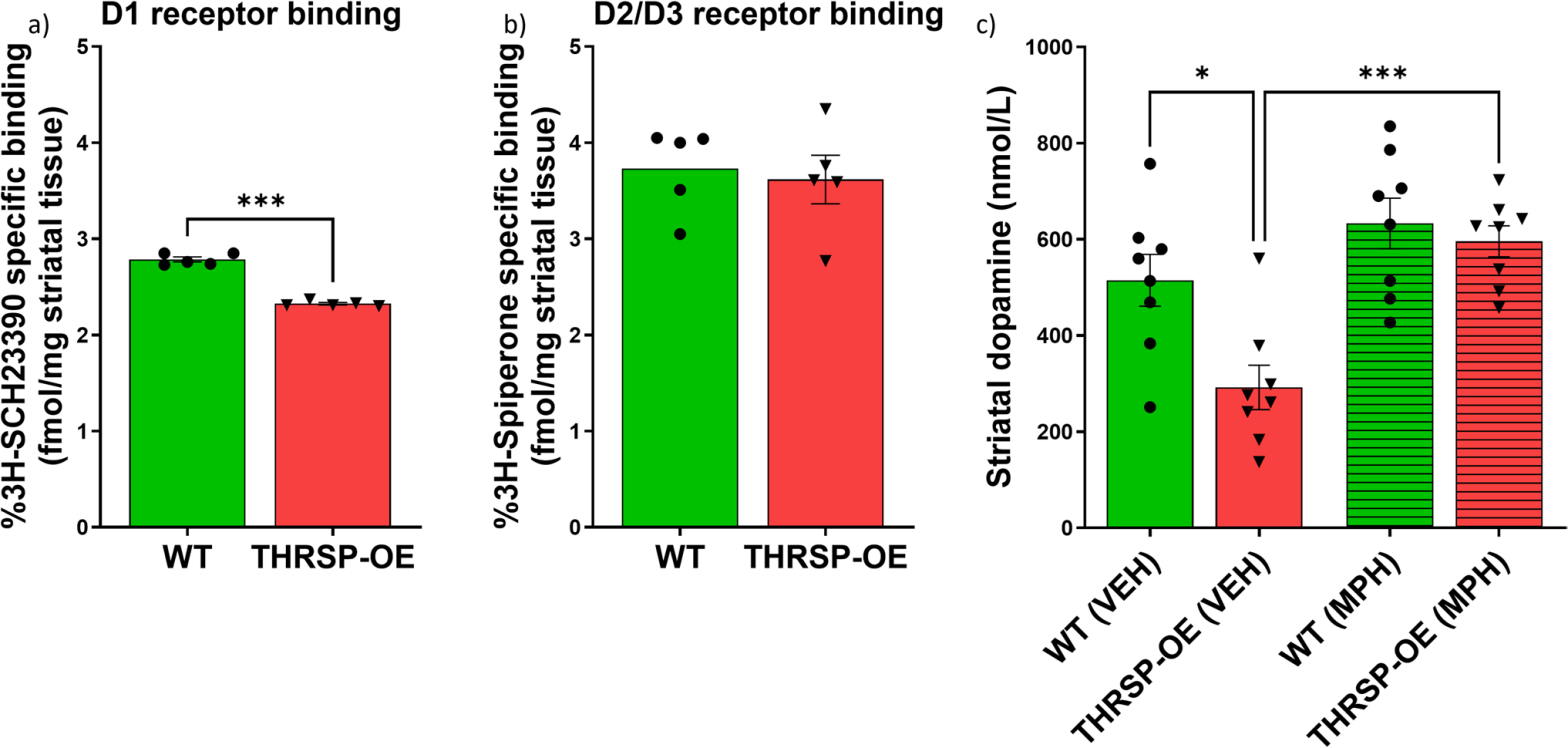
The dopamine receptor mediated signaling in THRSP-OE mice. Binding affinity towards dopamine D1 receptors and D2/3 receptors were analyzed in striatal samples of THRSP-OE mice (*n* = 5 samples/group; **a** unpaired *t*-test: *t* = 13.72, *df* = 4, *p* < 0.001; **b** unpaired *t*-test: *t* = 0.8739, *df* = 4, *p* = 0.4315). Moreover, dopamine levels in the striatum of mice treated with MPH (5 mg/kg) for 7 days were also analyzed (*n* = 8 samples/group; **c** one-way ANOVA, *F* (3, 28) = 10.6, *p* < 0.001). Values are presented as the mean ± standard error of the mean (SEM). *p < 0.05 ***p < 0.001, by unpaired *t*-test relative to WT and one-way ANOVA with Tukey’s multiple comparisons relative to vehicle (VEH)-treated WT and THRSP-OE mice

Dopamine neurotransmission plays a critical role in Snap25 function because dopamine is a key neurotransmitter in several important physiological processes, including reward, motivation, and movement. Dopamine is released into synapse by presynaptic neurons. It binds to specific receptors postsynaptic, activating downstream signaling pathways that regulate various cellular activities. The SNARE complex regulates dopamine release, which includes Snap25 as a key component [5, 35]. Snap25 plays a crucial role in the synaptic vesicular docking and fusion with the presynaptic membrane, allowing dopamine release into the synapse. Therefore, changes in Snap25 expression levels can have downstream effects on dopamine neurotransmission, potentially leading to alterations in behavior. Indeed, we found a reduced dopamine D1 receptor (D1R) binding (Fig. 7a) in THRSP-OE mice with no significant change in dopamine D2/D3 receptor (D2/3R) binding (Fig. 7b) relative to wild-type mice. Interestingly, this reduction in D1R binding was accompanied by low dopamine levels (Fig. 7c), which was rescued by methylphenidate (5 mg/kg) administration for seven days.

These findings show that the reduced D1R binding and low total striatal tissue dopamine concentrations in the striatum of the THRSP-OE may be a possible result of the altered function of the SNARE complex, leading to reduced dopamine release and signaling in the brain of THRSP-OE mice, which supports the ADHD-PI-like behaviors observed in this animal model. Additionally, a reduction in tyrosine 3-monooxygenase (commonly known as tyrosine hydroxylase), the rate-limiting enzyme in dopamine biosynthesis, may contribute to decreased dopamine levels by restricting its initial synthesis from L-tyrosine, further exacerbating the effects of impaired vesicular release. Notably, both THRSP-OE and THRSP-KO mice exhibit reduced expression of tyrosine 3-monooxygenase (see Table 1; DEP no. 36), with THRSP-OE mice showing a −2.2049-fold change (STN ratio), and THRSP-KO mice displaying an even more pronounced reduction (−4.2800 STN ratio). These findings suggest that THRSP dysregulation impairs dopamine synthesis regardless of whether THRSP is overexpressed or knocked out. However, it is important to note that the dopaminergic system in the brain is complex and involves multiple signaling pathways and receptors. In the case of ADHD, it has been proposed that there may be a dysfunction in the cortico-striatal networks, leading to the characteristic symptoms of the disorder [38–40]. Nonetheless, we have once again identified that THRSP-OE mice, an animal model for ADHD-PI, have impaired dopamine neurotransmission, as evidenced by low D1R binding and dopamine concentrations. Taken together, these results reinforce the relevance of THRSP-OE mice as a robust and mechanistically informative model for investigating the dopaminergic underpinnings of ADHDPI. However, continued investigation is warranted to fully elucidate how THRSP dysregulation alters dopamine-related molecular pathways and broader network-level processes underlying ADHD pathophysiology.

### EEG Changes in THRSP-OE Mice

Recent studies have used EEG to measure possible changes in brain wave activity to understand better the neural mechanisms underlying ADHD [41]. In line with this approach, the current study aimed to investigate EEG changes in early adult THRSP-OE mice, an animal model of ADHD-PI. Our previous finding identified EEG changes in the same strain at four weeks, which can be considered childhood age [8]. Therefore, the primary objective of this study is to report the EEG changes in THRSP-OE mice at an early adult age, which can facilitate a better understanding of the neural mechanisms underlying ADHD pathology between two crucial stages, childhood, and adulthood.

**Fig. 8:**
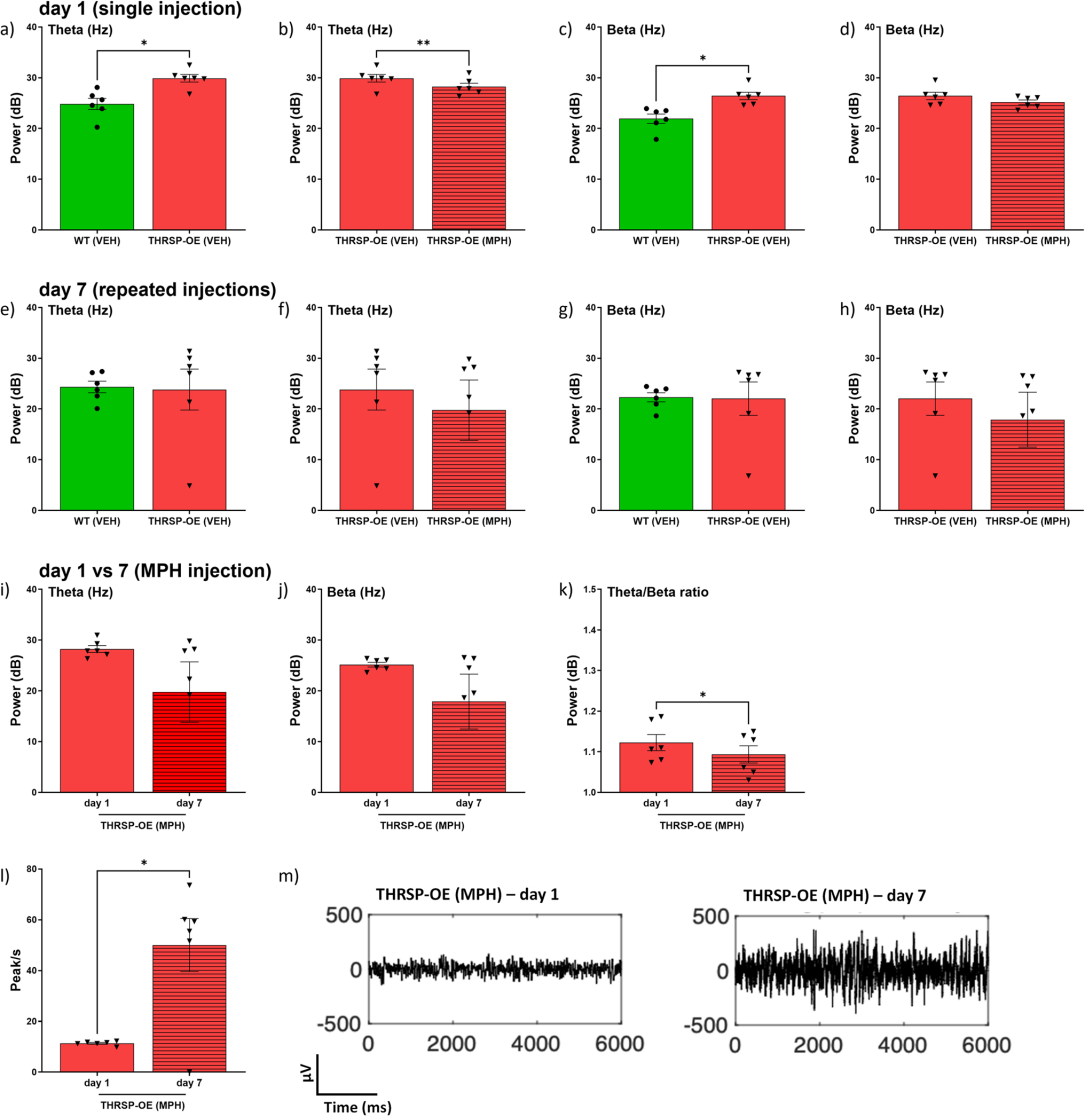
The EEG measurements (theta, beta) in THRSP-OE mice exposed to acute and repeated MPH (5 mg/kg). Mice (*n* = 6 samples/group) were monitored in an EEG apparatus after single (1 day) VEH and MPH administrations **a** Theta ((WT (VEH) × THRSP-OE (VEH); unpaired *t*-test, *t* = 3.23, *df* = 5, *p* = 0.023); **b** Theta ((THRSP (VEH) × THRSP-OE (MPH); unpaired *t*-test, *t* = 5.05, *df* = 5, *p* = 0.004); **c** Beta ((WT (VEH) × THRSP-OE (VEH); unpaired *t*-test, *t* = 2.76, *df* = 5, *p* = 0.040); **d** Beta ((THRSP (VEH) × THRSP-OE (VEH); unpaired *t*-test, *t* = 2.22, *df* = 5, *p* = 0.077), the repeated (7 days) VEH and MPH administrations; **e** Theta ((WT (VEH) × THRSP-OE (VEH); unpaired *t*-test, *t* = 109, *df* = 5, *p* = 0.918); **f** Theta ((THRSP (VEH) × THRSP-OE (MPH); unpaired *t*-test, *t* = 0.645, *df* = 5, *p* = 0.547); **g** Beta ((WT (VEH) × THRSP-OE (VEH); unpaired *t*-test, *t* = 0.0652, *df* = 5, *p* = 0.951); **h** Beta ((THRSP (VEH) × THRSP-OE (VEH); unpaired *t*-test, *t* = 0.767, *df* = 5, *p* = 0.478), and the comparison between 1 and 7 days of MPH effects in the EEG of THRSP-OE mice **i** Theta (THRSP-OE (MPH) (1 day × 7 days); paired *t*-test, *t* = 1.38, *df* = 5, *p* = 0.227); **j** Beta (THRSP-OE (MPH) (1 day × 7 days); paired *t*-test, *t* = 1.28, *df* = 5, *p* = 0.256); **k** Theta/Beta ratio (THRSP-OE (MPH) (1 day × 7 days); paired *t*-test, *t* = 2.72, *df* = 5, *p* = 0.042). **l** EEG peaks were also analyzed in THRSP-OE MPH-treated mice (1 day × 7 days); paired *t*-test, *t* = 3.71, *df* = 5, *p* = 0.014) and **m** graphical representations of the EEG amplitudes are also presented. Values are presented as the mean ± standard error of the mean (SEM). *p < 0.05 and **p < 0.01, by unpaired *t*-test relative to treatment and paired *t*-test for day effects

The study revealed significant findings: THRSP-OE mice exhibit innate high theta and beta waves (Fig. 8a, c) compared to the wild-type control. Acute methylphenidate administration was found to reduce the theta (Fig. 8b) but not beta waves (Fig. 8d) in mice. Interestingly, there were no changes in the theta and beta waves between THRSP-OE and wild-type mice during the 7 th day recording (Fig. 8e, g). Equally intriguing, there were no changes in theta or beta waves in the THRSP-OE mice between acute and repeated (7 days) dose of 5 mg/kg of methylphenidate administrations (Fig. 8f, h), although a slightly reduced theta and beta waves were observed, respectively, but failed to reach significance level. It is worth noting that our EEG study involved administering varying doses of methylphenidate (2, 5, and 10 mg/kg), but we primarily focused on the 5 mg/kg dose, as it was the only dose that yielded a significant difference between WT and THRSP-OE mice in our analysis. This finding is consistent with our previous research [6], which demonstrated that MPH administration at a dose of 5 mg/kg improves inattention and striatal dopamine-related gene (i.e., dopamine transporter, tyrosine hydroxylase, D1R, and D2R) expression in THRSP-OE mice. However, 7 days of methylphenidate administration increased the EEG peaks in THRSP-OE mice (Fig. 8l), which is an important finding. A representative frequency band was also shown (Fig. 8m). The observation of increased EEG peaks in THRSP-OE mice following 7 days of methylphenidate administration suggests that 5 mg/kg of methylphenidate may have a positive effect on the neural activity in THRSP-OE mice. This result is consistent with the known effects of methylphenidate on increasing neural activity and improving cognitive function in individuals with ADHD [42, 43]. Moreover, the theta and beta waves during the 1st and 7th day following methylphenidate administration (Fig. 8i, j) did not induce significant changes. However, the observation of reduced TBR is a significant finding in this ADHD-PI mouse model following 7 days of methylphenidate administration (Fig. 8k). Further, findings on the effects of methylphenidate in WT mice can also be found in Supplementary Fig. 1.

The TBR, a reliable measure of ADHD, is diagnosed based on the presence of a higher TBR in individuals with ADHD as compared to those without the disorder [17]. This ratio also resembles the pre-methylphenidate TBR in THRSP-OE mice (Fig. 8k), indicating the potential for improvements in TBR in this ADHD-PI animal model following 7 days of methylphenidate administration, a first-line drug for ADHD, a result supported by prior studies on the improvement of EEG spectral powers in children and adults with ADHD [44, 45]. In 2013, the US Food and Drug Administration (FDA) approved the use of EEG-based methods to diagnose ADHD, such as the Neuropsychiatric EEG-Based Assessment Aid (NEBA) System [46], a noninvasive neurophysiological scan test that gauges an increased brain oscillatory TBR in ADHD. These implications underscore the potential of our findings in advancing the understanding and treatment of ADHD.

Furthermore, our findings suggest that the THRSP-OE mouse model may be a useful tool for investigating the neural mechanisms underlying ADHD and the effects of methylphenidate administration and could potentially be used to inform the development of more effective treatments for ADHD in humans. The theta waves are associated with daydreaming and inattention, while beta waves are associated with focus and attention. Therefore, a higher ratio of theta to beta waves indicates greater inattention (and distractibility), a hallmark symptom of ADHD, and the specific symptom established and modeled by the THRSP-OE mice, an ADHD-PI animal model.

## Discussion

This extension study conducted in THRSP-OE mice, an ADHD-PI model, yielded some important results. Previous hippocampal proteomics studies indicated the involvement of the Wnt signaling pathway [7], and the current findings suggest potential dysfunctions in signal transduction and signaling due to possible impaired SNARE complex. This possible impairment in the SNARE complex may be attributed to the upregulation of the Snap25 protein found in the proteomics and the subsequent overexpression of the *snap25* gene observed. This innate overexpression of Snap25, a protein that is involved in regulating neurotransmitter release and is linked to ADHD [2] due to its involvement in dopamine signaling [5], maybe a contributing factor to the ADHD-PI-like behavior observed in THRSP-OE mice.

The research findings presented in this study shed light on the role of Snap25 protein or *snap25* gene expressions in the development of ADHD, which are consistent with previous studies in animal models and observations from mutations in Snap25 in humans, indicating that this protein plays a crucial role in inducing ADHD-like symptoms in animal models [28, 29] and ADHD in humans [2, 3, 5, 26, 27].

One such animal model is the Coloboma mutant (Cm/+) mouse, which is a highly validated ADHD transgenic mouse model [47] that was developed from neutron irradiation bearing a deletion mutation on chromosome 2, functionally disrupting approximately 20 genes, including *snap25* [4]. This mouse model was observed to have hyperactivity, inattention, and impulsivity due to the functional deletion of the *snap25* gene. However, when a transgene encoding snap25 was bred into the coloboma strain to complement the snap25 deletion, the ADHD symptoms expressed by these mice were rescued, returning their behaviors to normal levels as control [28]. This observation indicates the critical role of Snap25 protein in regulating neuronal activity and its potential implications in neuropsychiatric disorders like ADHD.

Interestingly, in another study [48], dopamine release in the dorsal striatum of the Cm/+ mouse was completely blocked, suggesting involvement in ADHD symptoms, particularly in the learning deficits observed in these mutants. This is quite an interesting comparison with THRSP-OE and Cm/+ mice since both have aberrant expressions of Snap25, in which the THRSP-OE mice were found to have upregulated Snap25 protein (or *snap25* gene) while the Cm/+ mice were found to have non-functioning *snap25* gene. However, while both have contrasting Snap25 expression levels, they share dysfunctional dopaminergic neurotransmission due to low DA levels in the striatum. While the Cm/+ mouse model has a non-functioning *snap25* gene, the THRSP-OE mouse model has upregulated Snap25 protein. This difference in *snap25* expression levels may indicate distinct underlying mechanisms for ADHD-like symptoms in these two models, warranting further investigation into the role of Snap25.

As previously mentioned, Snap25 is crucial in various enriched pathways, as shown in Table 3. These pathways range from synaptic vesicle trafficking to dopamine receptor-mediated signaling and thyrotropin-releasing hormone receptor signaling. This observation highlights the significant role of Snap25 proteins in these vital functions and may explain why the THRSP-OE mice behave as they do.

Of note, thyroid hormone (consisting of triiodothyronine (T3) and thyroxine (T4)) plays a crucial role in a variety of physiological processes. In particular, the brain, as part of the central nervous system, is a key target of thyroid hormones, which regulate neuronal cell proliferation, migration, and synaptogenesis [49]. Disruptions in thyroid hormone levels (such as hypothyroidism and GRTH) during fetal and postnatal periods can lead to developmental delays [50]. Furthermore, hypothyroidism in adulthood can cause neuronal network abnormalities, resulting in significant behavioral and neurological defects such as memory impairment and inattention [51]. Notably, several psychiatric disorders have been linked to TH abnormalities, including attention-deficit/hyperactivity disorder (ADHD) [52].

The THRSP-OE mice are models with overexpressed thyroid hormone-responsive protein (THRSP), a cofactor for thyroid hormone receptor beta (TRβ)-dependent transcriptional activation of specific gene targets [53]. This suggests that THRSP may modulate TRβ function downstream of the thyroid hormone response elements (TRE). However, it remains unclear how THRSP overexpression affects Snap25. Interestingly, a previous study has shown that thyroid hormone regulates the expression of Snap25 during rat brain development [54]. The study used microarray analysis and demonstrated that the Snap25 protein and *snap25* gene were down-regulated in the developing hypothyroid rat brain, indicating that hypothyroidism causes decreased Snap25 expression. This finding may account for the impaired brain development seen in hypothyroidism.

The findings of our study are particularly intriguing, as they suggest that there may be an interplay between Snap25, THRSP, and thyroid hormone signaling in the brain. This is highlighted by the fact that THRSP-OE mice have brain-specific (striatal) T3 deficiency [8], despite normal levels of circulating thyroid hormones, which raises important questions about the role of these genes in neural function, particularly in this mouse model of ADHD-PI. Nonetheless, our research indicates that the overexpression of THRSP in THRSP-OE mice leads to an innate overexpression of the striatal Snap25 protein. This, in turn, may cause downstream effects on multiple genes, pathways, and cellular functions, such as the SNARE complex and neurotransmitter release, leading to impaired dopamine signaling, as evidenced by low D1R binding and dopamine levels. These changes may also have contributed to altered EEG patterns observed in THRSP-OE mice, which were improved by methylphenidate administration. The upregulation of SNAP25 and other SNARE and VDCC-related genes in THRSP-OE mice may seem to contradict the expected increase in dopamine transmission. However, our findings suggest that the significant reductions in syt1 and munc13 gene expression may have downstream effects on the SNARE complex’s activity and the neurotransmitter release process, potentially leading to decreased dopamine transmission. This is consistent with the observed discrepancy between the protein and gene expression data, suggesting that post-transcriptional or post-translational modifications may be involved in regulating the expression or function of these proteins. Further studies are needed to fully understand the molecular mechanisms underlying the observed effects of snap25 gene overexpression in THRSP-OE mice on the SNARE complex and neurotransmitter release, particularly dopamine.

Our findings also suggest that the changes in dopamine receptor-mediated signaling in THRSP-OE mice may be related to the altered expression of Snap25 and other proteins involved in this pathway, which could potentially affect dopamine levels and neurotransmission. The reduction in tyrosine 3-monooxygenase (Th) levels in THRSP-OE mice, as shown in Table 3, further supports this idea. Th, also known as tyrosine hydroxylase, is an enzyme involved in the biosynthesis of dopamine, and its reduced expression in THRSP-OE mice may contribute to the impaired dopamine signaling observed in these mice. These findings highlight the complex interplay between Snap25, Th, and other proteins involved in dopamine receptor-mediated signaling, and suggest that further studies are needed to fully understand the mechanisms underlying these changes.

However, it is important to note that there are limitations to this study that must be considered when interpreting our findings. Specifically, while we observed alterations in dopamine levels and SNARE protein expression, we did not directly assess dopamine release or turnover in THRSP-OE mice. As such, while the observed changes in dopamine signaling are consistent with our hypothesis, the lack of direct measurement of dopamine release limits the ability to conclusively determine the impact of Snap25 overexpression on dopamine neurotransmission. The proposed mechanisms linking altered SNARE function and dopamine signaling remain speculative and will require further experimental validation. Future studies that directly measure dopamine release, either *in vivo* or *ex vivo*, or assess dopamine turnover, will be essential to confirm these findings and clarify the molecular mechanisms involved.

Furthermore, research has shown that ADHD is associated with changes in both neurotransmitter activity and EEG patterns. Poor dopaminergic signaling is believed to be a key contributing factor to these changes, as dopamine plays a crucial role in regulating attention, movement, and motivation. In individuals with ADHD, there is often a reduction in dopamine levels, which can lead to difficulties in paying attention and regulating behavior. Studies have shown that there are characteristic EEG patterns associated with ADHD, including increased TBR, which may be related to the underlying neurotransmitter imbalances in ADHD.

Overall, our findings underscore the complex interplay between gene/protein factors, neurotransmitter systems, and neural cicuitry in THRSP-OE mice, a valuable model for ADHD-PI. These results emphasize the potential role of THRSP dysregulation in modulating dopamine-related mechanisms and synaptic function underlying ADHD-like behaviors. Continued research is needed to further clarify the role of THRSP and associated pathways in ADHD pathophysiology and to leverage the THRSP-OE model in the development of more targeted and effective therapeutic strategies, particularly for adults affected by this disorder.

## Supplementary Information

Below is the link to the electronic supplementary material.

Supplementary Figure 1 (TIF 1147 KB)

Supplementary Table 1 (DOCX 21 KB)

Supplementary Table 2 (DOCX 19 KB)

Supplementary Table 3 (DOCX 23 KB)

Supplementary Table 4 (DOCX 22 KB)

Supplementary Table 5 (DOCX 16 KB)

## Data Availability

Proteomics data are available via ProteomeXchange with identifier PXD051619.
