# Supplementary figures and images for: Unraveling Predominantly Inattentive ADHD (ADHD-PI): Insights from Proteomic Analysis of the Striatum of Thyroid Hormone-Responsive Protein (THRSP)–Overexpressing Mice
Source: Mol Neurobiol. 2025 Jun 10;62(10):13225–49. doi: 10.1007/s12035-025-05031-z (PMC12433356; doi:10.1007/s12035-025-05031-z)

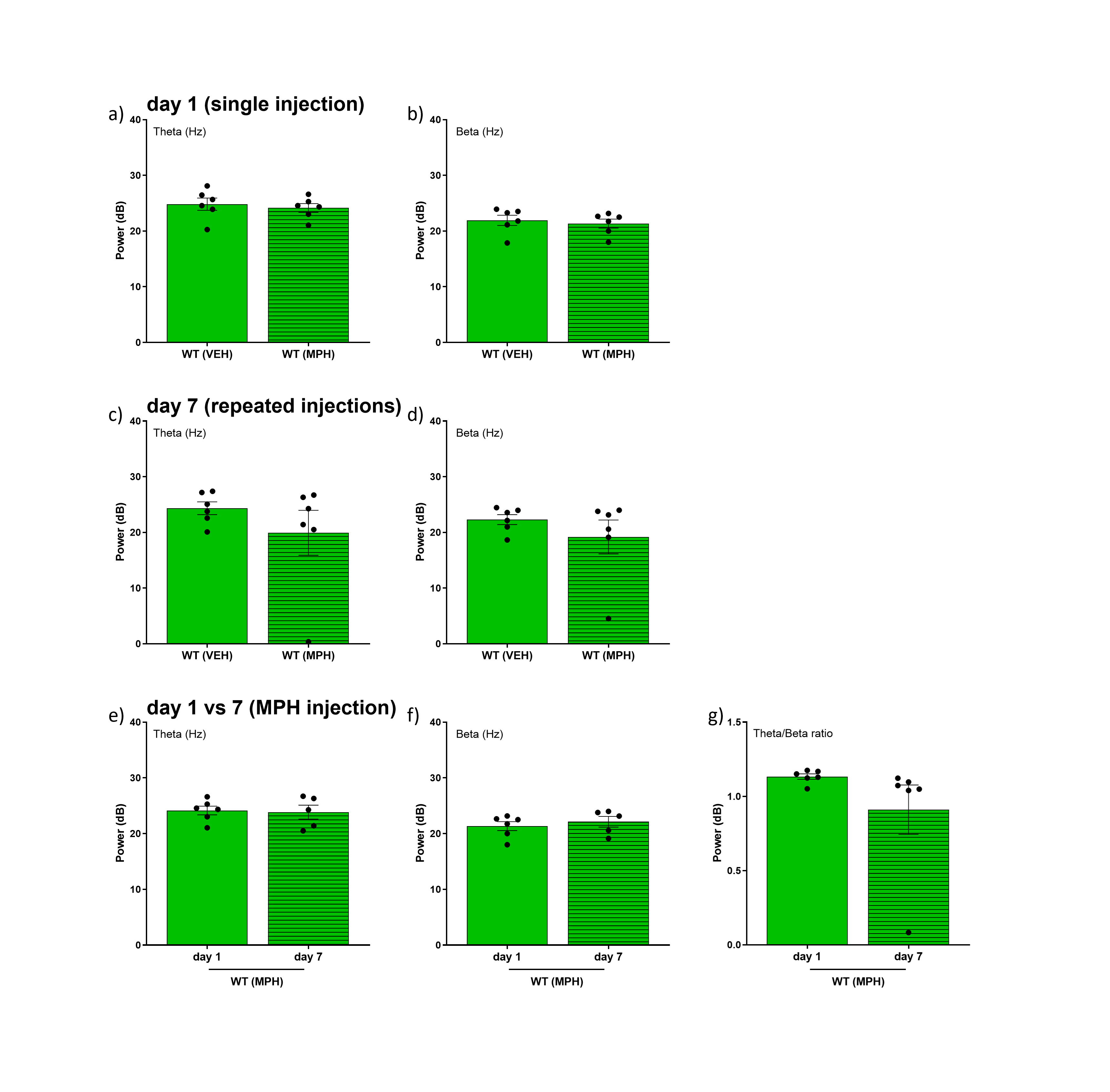

Supplement: Supplementary file 1 — Supplementary Figure 1 (TIF 1147 KB) [file 12035_2025_5031_MOESM1_ESM.tif]
