# Supplementary material for: Unraveling Predominantly Inattentive ADHD (ADHD-PI): Insights from Proteomic Analysis of the Striatum of Thyroid Hormone-Responsive Protein (THRSP)–Overexpressing Mice
Source: Mol Neurobiol. 2025 Jun 10;62(10):13225–49. doi: 10.1007/s12035-025-05031-z (PMC12433356; doi:10.1007/s12035-025-05031-z)
Supplement: Supplementary file 2 — Supplementary Table 1 (DOCX 21 KB) [file 12035_2025_5031_MOESM2_ESM.docx]

Supplementary Table 1. Complete list of PANTHER GO biological processes identified from the upregulated proteins in THRSP-OE mice.

| **PANTHER GO Biological Process** | **Mus musculus - Reference list (21983)** | **THRSP-OE** | | | |
| --- | --- | --- | --- | --- | --- |
|  |  | **Upregulated proteins (32)** | **Upregulated proteins (over/under)** | **Upregulated proteins (fold Enrichment)** | **Upregulated proteins (FDR; p-value)** |
| vesicle fusion to plasma membrane (GO:0099500) | 10 | 3 | + | > 100 | 0.0001 |
| exocytic process (GO:0140029) | 29 | 6 | + | > 100 | 0.0000 |
| vesicle fusion (GO:0006906) | 52 | 9 | + | > 100 | 0.0000 |
| organelle membrane fusion (GO:0090174) | 53 | 9 | + | > 100 | 0.0000 |
| organelle fusion (GO:0048284) | 68 | 9 | + | 90.92 | 0.0000 |
| synaptic vesicle exocytosis (GO:0016079) | 32 | 4 | + | 85.87 | 0.0000 |
| membrane fusion (GO:0061025) | 74 | 9 | + | 83.55 | 0.0000 |
| establishment of protein localization to extracellular region (GO:0035592) | 26 | 3 | + | 79.27 | 0.0006 |
| protein secretion (GO:0009306) | 26 | 3 | + | 79.27 | 0.0006 |
| protein localization to extracellular region (GO:0071692) | 26 | 3 | + | 79.27 | 0.0006 |
| regulated exocytosis (GO:0045055) | 45 | 4 | + | 61.06 | 0.0001 |
| neurotransmitter secretion (GO:0007269) | 46 | 4 | + | 59.74 | 0.0001 |
| signal release from synapse (GO:0099643) | 46 | 4 | + | 59.74 | 0.0001 |
| vesicle organization (GO:0016050) | 114 | 9 | + | 54.23 | 0.0000 |
| signal release (GO:0023061) | 53 | 4 | + | 51.85 | 0.0001 |
| neurotransmitter transport (GO:0006836) | 55 | 4 | + | 49.96 | 0.0001 |
| exocytosis (GO:0006887) | 92 | 6 | + | 44.8 | 0.0000 |
| regulation of neurotransmitter levels (GO:0001505) | 64 | 4 | + | 42.94 | 0.0002 |
| regulation of exocytosis (GO:0017157) | 67 | 4 | + | 41.01 | 0.0003 |
| synaptic vesicle cycle (GO:0099504) | 68 | 4 | + | 40.41 | 0.0003 |
| vesicle-mediated transport in synapse (GO:0099003) | 68 | 4 | + | 40.41 | 0.0003 |
| protein localization to cell periphery (GO:1990778) | 74 | 4 | + | 37.13 | 0.0003 |
| secretion by cell (GO:0032940) | 118 | 6 | + | 34.93 | 0.0000 |
| secretion (GO:0046903) | 121 | 6 | + | 34.06 | 0.0000 |
| protein localization to plasma membrane (GO:0072659) | 64 | 3 | + | 32.2 | 0.0051 |
| export from cell (GO:0140352) | 137 | 6 | + | 30.09 | 0.0000 |
| regulation of vesicle-mediated transport (GO:0060627) | 115 | 5 | + | 29.87 | 0.0001 |
| regulation of secretion by cell (GO:1903530) | 95 | 4 | + | 28.92 | 0.0007 |
| membrane organization (GO:0061024) | 219 | 9 | + | 28.23 | 0.0000 |
| organelle localization (GO:0051640) | 150 | 5 | + | 22.9 | 0.0002 |
| regulation of secretion (GO:0051046) | 123 | 4 | + | 22.34 | 0.0016 |
| endomembrane system organization (GO:0010256) | 165 | 5 | + | 20.82 | 0.0003 |
| chemical synaptic transmission (GO:0007268) | 177 | 5 | + | 19.41 | 0.0004 |
| anterograde trans-synaptic signaling (GO:0098916) | 177 | 5 | + | 19.41 | 0.0004 |
| trans-synaptic signaling (GO:0099537) | 180 | 5 | + | 19.08 | 0.0004 |
| protein localization to membrane (GO:0072657) | 152 | 4 | + | 18.08 | 0.0033 |
| synaptic signaling (GO:0099536) | 198 | 5 | + | 17.35 | 0.0006 |
| vesicle-mediated transport (GO:0016192) | 556 | 14 | + | 17.3 | 0.0000 |
| localization within membrane (GO:0051668) | 189 | 4 | + | 14.54 | 0.0066 |
| Golgi vesicle transport (GO:0048193) | 147 | 3 | + | 14.02 | 0.0473 |
| protein-containing complex assembly (GO:0065003) | 270 | 5 | + | 12.72 | 0.0021 |
| cell-cell signaling (GO:0007267) | 292 | 5 | + | 11.76 | 0.0030 |
| regulation of transport (GO:0051049) | 297 | 5 | + | 11.57 | 0.0031 |
| protein transport (GO:0015031) | 368 | 6 | + | 11.2 | 0.0008 |
| regulation of localization (GO:0032879) | 372 | 6 | + | 11.08 | 0.0008 |
| establishment of protein localization (GO:0045184) | 381 | 6 | + | 10.82 | 0.0009 |
| establishment of localization in cell (GO:0051649) | 760 | 10 | + | 9.04 | 0.0000 |
| protein-containing complex organization (GO:0043933) | 413 | 5 | + | 8.32 | 0.0119 |
| cellular localization (GO:0051641) | 978 | 11 | + | 7.73 | 0.0000 |
| nitrogen compound transport (GO:0071705) | 541 | 6 | + | 7.62 | 0.0051 |
| cellular macromolecule localization (GO:0070727) | 553 | 6 | + | 7.45 | 0.0054 |
| protein localization (GO:0008104) | 553 | 6 | + | 7.45 | 0.0053 |
| intracellular transport (GO:0046907) | 651 | 7 | + | 7.39 | 0.0017 |
| regulation of biological quality (GO:0065008) | 742 | 7 | + | 6.48 | 0.0036 |
| transport (GO:0006810) | 1699 | 16 | + | 6.47 | 0.0000 |
| establishment of localization (GO:0051234) | 1738 | 16 | + | 6.32 | 0.0000 |
| localization (GO:0051179) | 1911 | 16 | + | 5.75 | 0.0000 |
| macromolecule localization (GO:0033036) | 736 | 6 | + | 5.6 | 0.0226 |
| organelle organization (GO:0006996) | 1306 | 10 | + | 5.26 | 0.0006 |
| organic substance transport (GO:0071702) | 813 | 6 | + | 5.07 | 0.0372 |
| cellular component organization (GO:0016043) | 2110 | 11 | + | 3.58 | 0.0051 |
| cellular component organization or biogenesis (GO:0071840) | 2233 | 11 | + | 3.38 | 0.0077 |
