# Supplementary material for: Unraveling Predominantly Inattentive ADHD (ADHD-PI): Insights from Proteomic Analysis of the Striatum of Thyroid Hormone-Responsive Protein (THRSP)–Overexpressing Mice
Source: Mol Neurobiol. 2025 Jun 10;62(10):13225–49. doi: 10.1007/s12035-025-05031-z (PMC12433356; doi:10.1007/s12035-025-05031-z)
Supplement: Supplementary file 3 — Supplementary Table 2 (DOCX 19 KB) [file 12035_2025_5031_MOESM3_ESM.docx]

Supplementary Table 2. Complete list of PANTHER GO biological processes identified from the downregulated proteins in THRSP-OE mice.

| **PANTHER GO Biological Process** | **Mus musculus - Reference list (21983)** | **THRSP-OE** | | | |
| --- | --- | --- | --- | --- | --- |
|  |  | **Downregulated proteins (35)** | **Downregulated proteins (over/under)** | **Downregulated proteins (fold Enrichment)** | **Downregulated proteins (FDR; p-value)** |
| neurotransmitter secretion (GO:0007269) | 46 | 3 | + | 40.96 | 0.0129 |
| signal release from synapse (GO:0099643) | 46 | 3 | + | 40.96 | 0.0118 |
| signal release (GO:0023061) | 53 | 3 | + | 35.55 | 0.0151 |
| neurotransmitter transport (GO:0006836) | 55 | 3 | + | 34.26 | 0.0147 |
| regulation of neurotransmitter levels (GO:0001505) | 64 | 3 | + | 29.44 | 0.0213 |
| secretion by cell (GO:0032940) | 118 | 4 | + | 21.29 | 0.0129 |
| secretion (GO:0046903) | 121 | 4 | + | 20.76 | 0.0125 |
| exocytosis (GO:0006887) | 92 | 3 | + | 20.48 | 0.0461 |
| regulation of secretion by cell (GO:1903530) | 95 | 3 | + | 19.83 | 0.0483 |
| export from cell (GO:0140352) | 137 | 4 | + | 18.34 | 0.0123 |
| chemical synaptic transmission (GO:0007268) | 177 | 5 | + | 17.74 | 0.0214 |
| anterograde trans-synaptic signaling (GO:0098916) | 177 | 5 | + | 17.74 | 0.0107 |
| trans-synaptic signaling (GO:0099537) | 180 | 5 | + | 17.45 | 0.0077 |
| synaptic signaling (GO:0099536) | 198 | 5 | + | 15.86 | 0.0091 |
| cell-cell signaling (GO:0007267) | 292 | 5 | + | 10.75 | 0.0148 |
| regulation of localization (GO:0032879) | 372 | 5 | + | 8.44 | 0.0320 |
| vesicle-mediated transport (GO:0016192) | 556 | 6 | + | 6.78 | 0.0270 |
| establishment of localization in cell (GO:0051649) | 760 | 7 | + | 5.79 | 0.0213 |
| cellular localization (GO:0051641) | 978 | 9 | + | 5.78 | 0.0076 |
| establishment of localization (GO:0051234) | 1738 | 11 | + | 3.98 | 0.0131 |
| localization (GO:0051179) | 1911 | 12 | + | 3.94 | 0.0087 |
| transport (GO:0006810) | 1699 | 10 | + | 3.7 | 0.0258 |
| cellular process (GO:0009987) | 8229 | 25 | + | 1.91 | 0.0141 |
