# Supplementary material for: Unraveling Predominantly Inattentive ADHD (ADHD-PI): Insights from Proteomic Analysis of the Striatum of Thyroid Hormone-Responsive Protein (THRSP)–Overexpressing Mice
Source: Mol Neurobiol. 2025 Jun 10;62(10):13225–49. doi: 10.1007/s12035-025-05031-z (PMC12433356; doi:10.1007/s12035-025-05031-z)
Supplement: Supplementary file 4 — Supplementary Table 3 (DOCX 23 KB) [file 12035_2025_5031_MOESM4_ESM.docx]

Supplementary Table 3. Complete list of PANTHER GO biological processes identified from the upregulated proteins in THRSP-KO mice.

| **PANTHER GO Biological Process** | **Mus musculus - Reference list (21983)** | **THRSP-KO** | | | |
| --- | --- | --- | --- | --- | --- |
|  |  | **Upregulated proteins (23)** | **Upregulated proteins (over/under)** | **Upregulated proteins (fold Enrichment)** | **Upregulated proteins (FDR; p-value)** |
| sperm axoneme assembly (GO:0007288) | 3 | 2 | + | > 100 | 0.0007 |
| epithelial cilium movement involved in extracellular fluid movement (GO:0003351) | 3 | 2 | + | > 100 | 0.0007 |
| filopodium assembly (GO:0046847) | 7 | 2 | + | > 100 | 0.0022 |
| motile cilium assembly (GO:0044458) | 9 | 2 | + | > 100 | 0.0032 |
| neurotransmitter receptor transport to postsynaptic membrane (GO:0098969) | 9 | 2 | + | > 100 | 0.0031 |
| neuron projection extension (GO:1990138) | 10 | 2 | + | > 100 | 0.0037 |
| developmental cell growth (GO:0048588) | 10 | 2 | + | > 100 | 0.0036 |
| cell growth (GO:0016049) | 10 | 2 | + | > 100 | 0.0035 |
| developmental growth involved in morphogenesis (GO:0060560) | 10 | 2 | + | > 100 | 0.0034 |
| establishment of protein localization to extracellular region (GO:0035592) | 26 | 5 | + | > 100 | 0.0000 |
| protein secretion (GO:0009306) | 26 | 5 | + | > 100 | 0.0000 |
| protein localization to extracellular region (GO:0071692) | 26 | 5 | + | > 100 | 0.0000 |
| exocytic process (GO:0140029) | 29 | 5 | + | > 100 | 0.0000 |
| sperm motility (GO:0097722) | 14 | 2 | + | > 100 | 0.0059 |
| flagellated sperm motility (GO:0030317) | 14 | 2 | + | > 100 | 0.0057 |
| protein localization to synapse (GO:0035418) | 14 | 2 | + | > 100 | 0.0056 |
| developmental growth (GO:0048589) | 16 | 2 | + | > 100 | 0.0070 |
| growth (GO:0040007) | 16 | 2 | + | > 100 | 0.0069 |
| regulation of postsynaptic membrane neurotransmitter receptor levels (GO:0099072) | 17 | 2 | + | > 100 | 0.0076 |
| endocytic recycling (GO:0032456) | 31 | 3 | + | 88.64 | 0.0004 |
| protein localization to cell junction (GO:1902414) | 21 | 2 | + | 87.23 | 0.0106 |
| regulation of endocytosis (GO:0030100) | 32 | 3 | + | 85.87 | 0.0005 |
| regulation of exocytosis (GO:0017157) | 67 | 6 | + | 82.03 | 0.0000 |
| regulation of vesicle-mediated transport (GO:0060627) | 115 | 9 | + | 71.68 | 0.0000 |
| vesicle fusion (GO:0006906) | 52 | 4 | + | 70.46 | 0.0000 |
| organelle membrane fusion (GO:0090174) | 53 | 4 | + | 69.13 | 0.0000 |
| vesicle-mediated transport to the plasma membrane (GO:0098876) | 54 | 4 | + | 67.85 | 0.0000 |
| axoneme assembly (GO:0035082) | 29 | 2 | + | 63.17 | 0.0188 |
| protein localization to cell periphery (GO:1990778) | 74 | 5 | + | 61.89 | 0.0000 |
| regulation of secretion by cell (GO:1903530) | 95 | 6 | + | 57.85 | 0.0000 |
| protein localization to plasma membrane (GO:0072659) | 64 | 4 | + | 57.25 | 0.0001 |
| organelle fusion (GO:0048284) | 68 | 4 | + | 53.88 | 0.0001 |
| regulation of secretion (GO:0051046) | 123 | 7 | + | 52.13 | 0.0000 |
| exocytosis (GO:0006887) | 92 | 5 | + | 49.78 | 0.0000 |
| membrane fusion (GO:0061025) | 74 | 4 | + | 49.51 | 0.0001 |
| cilium movement involved in cell motility (GO:0060294) | 39 | 2 | + | 46.97 | 0.0291 |
| secretion by cell (GO:0032940) | 118 | 6 | + | 46.57 | 0.0000 |
| microtubule bundle formation (GO:0001578) | 40 | 2 | + | 45.8 | 0.0301 |
| secretion (GO:0046903) | 121 | 6 | + | 45.42 | 0.0000 |
| cilium or flagellum-dependent cell motility (GO:0001539) | 44 | 2 | + | 41.63 | 0.0351 |
| cilium-dependent cell motility (GO:0060285) | 44 | 2 | + | 41.63 | 0.0346 |
| export from cell (GO:0140352) | 137 | 6 | + | 40.11 | 0.0000 |
| signal release (GO:0023061) | 53 | 2 | + | 34.56 | 0.0486 |
| cilium movement (GO:0003341) | 53 | 2 | + | 34.56 | 0.0480 |
| vesicle organization (GO:0016050) | 114 | 4 | + | 32.14 | 0.0005 |
| regulation of transport (GO:0051049) | 297 | 10 | + | 30.84 | 0.0000 |
| organelle localization (GO:0051640) | 150 | 5 | + | 30.53 | 0.0001 |
| protein localization to membrane (GO:0072657) | 152 | 5 | + | 30.13 | 0.0001 |
| regulation of localization (GO:0032879) | 372 | 10 | + | 24.62 | 0.0000 |
| localization within membrane (GO:0051668) | 189 | 5 | + | 24.23 | 0.0001 |
| endosomal transport (GO:0016197) | 115 | 3 | + | 23.89 | 0.0106 |
| Golgi vesicle transport (GO:0048193) | 147 | 3 | + | 18.69 | 0.0201 |
| cilium assembly (GO:0060271) | 151 | 3 | + | 18.2 | 0.0214 |
| microtubule-based movement (GO:0007018) | 164 | 3 | + | 16.76 | 0.0266 |
| plasma membrane bounded cell projection assembly (GO:0120031) | 164 | 3 | + | 16.76 | 0.0262 |
| membrane organization (GO:0061024) | 219 | 4 | + | 16.73 | 0.0042 |
| cilium organization (GO:0044782) | 172 | 3 | + | 15.98 | 0.0291 |
| cell projection assembly (GO:0030031) | 173 | 3 | + | 15.88 | 0.0291 |
| protein transport (GO:0015031) | 368 | 6 | + | 14.93 | 0.0002 |
| vesicle-mediated transport (GO:0016192) | 556 | 9 | + | 14.83 | 0.0000 |
| establishment of protein localization (GO:0045184) | 381 | 6 | + | 14.42 | 0.0002 |
| nitrogen compound transport (GO:0071705) | 541 | 6 | + | 10.16 | 0.0012 |
| cellular macromolecule localization (GO:0070727) | 553 | 6 | + | 9.94 | 0.0014 |
| protein localization (GO:0008104) | 553 | 6 | + | 9.94 | 0.0013 |
| establishment of localization in cell (GO:0051649) | 760 | 8 | + | 9.64 | 0.0001 |
| regulation of cellular component organization (GO:0051128) | 416 | 4 | + | 8.81 | 0.0319 |
| intracellular transport (GO:0046907) | 651 | 6 | + | 8.44 | 0.0031 |
| cellular localization (GO:0051641) | 978 | 9 | + | 8.43 | 0.0000 |
| macromolecule localization (GO:0033036) | 736 | 6 | + | 7.47 | 0.0051 |
| transport (GO:0006810) | 1699 | 13 | + | 7.01 | 0.0000 |
| establishment of localization (GO:0051234) | 1738 | 13 | + | 6.85 | 0.0000 |
| organic substance transport (GO:0071702) | 813 | 6 | + | 6.76 | 0.0076 |
| localization (GO:0051179) | 1911 | 13 | + | 6.23 | 0.0000 |
| organelle organization (GO:0006996) | 1306 | 7 | + | 4.91 | 0.0137 |
| biological regulation (GO:0065007) | 6264 | 15 | + | 2.19 | 0.0286 |
