# Supplementary material for: Unraveling Predominantly Inattentive ADHD (ADHD-PI): Insights from Proteomic Analysis of the Striatum of Thyroid Hormone-Responsive Protein (THRSP)–Overexpressing Mice
Source: Mol Neurobiol. 2025 Jun 10;62(10):13225–49. doi: 10.1007/s12035-025-05031-z (PMC12433356; doi:10.1007/s12035-025-05031-z)
Supplement: Supplementary file 5 — Supplementary Table 4 (DOCX 22 KB) [file 12035_2025_5031_MOESM5_ESM.docx]

Supplementary Table 4. Complete list of PANTHER GO biological processes identified from the downregulated proteins in THRSP-KO mice.

| **PANTHER GO Biological Process** | **Mus musculus - Reference list (21983)** | **THRSP-KO** | | | |
| --- | --- | --- | --- | --- | --- |
|  |  | **Downregulated proteins (69)** | **Downregulated proteins (over/under)** | **Downregulated proteins (fold Enrichment)** | **Downregulated proteins (FDR; p-value)** |
| neuromuscular junction development (GO:0007528) | 9 | 3 | + | > 100 | 0.0004 |
| synaptic transmission, glutamatergic (GO:0035249) | 20 | 5 | + | 78.51 | 0.0000 |
| receptor clustering (GO:0043113) | 9 | 2 | + | 69.79 | 0.0191 |
| calcium-ion regulated exocytosis (GO:0017156) | 23 | 5 | + | 68.27 | 0.0000 |
| vesicle fusion to plasma membrane (GO:0099500) | 10 | 2 | + | 62.81 | 0.0225 |
| clathrin-dependent endocytosis (GO:0072583) | 16 | 3 | + | 58.88 | 0.0013 |
| synaptic vesicle exocytosis (GO:0016079) | 32 | 6 | + | 58.88 | 0.0000 |
| neurotransmitter secretion (GO:0007269) | 46 | 8 | + | 54.62 | 0.0000 |
| signal release from synapse (GO:0099643) | 46 | 8 | + | 54.62 | 0.0000 |
| exocytic process (GO:0140029) | 29 | 5 | + | 54.15 | 0.0000 |
| synaptic vesicle endocytosis (GO:0048488) | 32 | 5 | + | 49.07 | 0.0000 |
| regulated exocytosis (GO:0045055) | 45 | 7 | + | 48.85 | 0.0000 |
| establishment or maintenance of epithelial cell apical/basal polarity (GO:0045197) | 13 | 2 | + | 48.31 | 0.0344 |
| signal release (GO:0023061) | 53 | 8 | + | 47.4 | 0.0000 |
| synaptic vesicle recycling (GO:0036465) | 34 | 5 | + | 46.18 | 0.0000 |
| synaptic vesicle cycle (GO:0099504) | 68 | 10 | + | 46.18 | 0.0000 |
| vesicle-mediated transport in synapse (GO:0099003) | 68 | 10 | + | 46.18 | 0.0000 |
| neurotransmitter transport (GO:0006836) | 55 | 8 | + | 45.68 | 0.0000 |
| establishment or maintenance of bipolar cell polarity (GO:0061245) | 14 | 2 | + | 44.86 | 0.0381 |
| establishment or maintenance of apical/basal cell polarity (GO:0035088) | 14 | 2 | + | 44.86 | 0.0375 |
| regulation of neurotransmitter levels (GO:0001505) | 64 | 9 | + | 44.16 | 0.0000 |
| receptor-mediated endocytosis (GO:0006898) | 49 | 5 | + | 32.05 | 0.0000 |
| synapse organization (GO:0050808) | 55 | 5 | + | 28.55 | 0.0001 |
| phagocytosis (GO:0006909) | 34 | 3 | + | 27.71 | 0.0087 |
| chemical synaptic transmission (GO:0007268) | 177 | 14 | + | 24.84 | 0.0000 |
| anterograde trans-synaptic signaling (GO:0098916) | 177 | 14 | + | 24.84 | 0.0000 |
| trans-synaptic signaling (GO:0099537) | 180 | 14 | + | 24.43 | 0.0000 |
| vesicle fusion (GO:0006906) | 52 | 4 | + | 24.16 | 0.0014 |
| secretion by cell (GO:0032940) | 118 | 9 | + | 23.95 | 0.0000 |
| exocytosis (GO:0006887) | 92 | 7 | + | 23.89 | 0.0000 |
| synaptic signaling (GO:0099536) | 198 | 15 | + | 23.79 | 0.0000 |
| organelle membrane fusion (GO:0090174) | 53 | 4 | + | 23.7 | 0.0014 |
| secretion (GO:0046903) | 121 | 9 | + | 23.36 | 0.0000 |
| endocytosis (GO:0006897) | 154 | 11 | + | 22.43 | 0.0000 |
| vesicle organization (GO:0016050) | 114 | 8 | + | 22.04 | 0.0000 |
| export from cell (GO:0140352) | 137 | 9 | + | 20.63 | 0.0000 |
| organelle fusion (GO:0048284) | 68 | 4 | + | 18.47 | 0.0034 |
| membrane fusion (GO:0061025) | 74 | 4 | + | 16.98 | 0.0045 |
| regulation of trans-synaptic signaling (GO:0099177) | 93 | 5 | + | 16.88 | 0.0008 |
| modulation of chemical synaptic transmission (GO:0050804) | 93 | 5 | + | 16.88 | 0.0008 |
| regulation of secretion by cell (GO:1903530) | 95 | 5 | + | 16.53 | 0.0008 |
| cell-cell signaling (GO:0007267) | 292 | 15 | + | 16.13 | 0.0000 |
| regulation of exocytosis (GO:0017157) | 67 | 3 | + | 14.06 | 0.0460 |
| import into cell (GO:0098657) | 246 | 11 | + | 14.04 | 0.0000 |
| regulation of secretion (GO:0051046) | 123 | 5 | + | 12.77 | 0.0024 |
| organelle localization (GO:0051640) | 150 | 6 | + | 12.56 | 0.0006 |
| cell junction organization (GO:0034330) | 142 | 5 | + | 11.06 | 0.0043 |
| vesicle-mediated transport (GO:0016192) | 556 | 19 | + | 10.73 | 0.0000 |
| membrane organization (GO:0061024) | 219 | 7 | + | 10.04 | 0.0004 |
| establishment of localization in cell (GO:0051649) | 760 | 17 | + | 7.02 | 0.0000 |
| regulation of biological quality (GO:0065008) | 742 | 16 | + | 6.77 | 0.0000 |
| regulation of localization (GO:0032879) | 372 | 8 | + | 6.75 | 0.0013 |
| cellular localization (GO:0051641) | 978 | 20 | + | 6.42 | 0.0000 |
| transport (GO:0006810) | 1699 | 28 | + | 5.18 | 0.0000 |
| establishment of localization (GO:0051234) | 1738 | 28 | + | 5.06 | 0.0000 |
| localization (GO:0051179) | 1911 | 30 | + | 4.93 | 0.0000 |
| regulation of cell communication (GO:0010646) | 706 | 9 | + | 4 | 0.0159 |
| regulation of signaling (GO:0023051) | 709 | 9 | + | 3.99 | 0.0161 |
| organelle organization (GO:0006996) | 1306 | 14 | + | 3.37 | 0.0025 |
| signaling (GO:0023052) | 2267 | 20 | + | 2.77 | 0.0009 |
| cell communication (GO:0007154) | 2307 | 20 | + | 2.72 | 0.0011 |
| cellular component organization (GO:0016043) | 2110 | 17 | + | 2.53 | 0.0107 |
| cellular component organization or biogenesis (GO:0071840) | 2233 | 17 | + | 2.39 | 0.0193 |
| cellular process (GO:0009987) | 8229 | 48 | + | 1.83 | 0.0000 |
| biological regulation (GO:0065007) | 6264 | 34 | + | 1.7 | 0.0169 |
| regulation of biological process (GO:0050789) | 5966 | 32 | + | 1.68 | 0.0350 |
| biological_process (GO:0008150) | 12919 | 59 | + | 1.43 | 0.0004 |
| Unclassified (UNCLASSIFIED) | 9064 | 11 | - | 0.38 | 0.0004 |
| regulation of gene expression (GO:0010468) | 2311 | 0 | - | < 0.01 | 0.0229 |
