# Supplementary material for: Unraveling Predominantly Inattentive ADHD (ADHD-PI): Insights from Proteomic Analysis of the Striatum of Thyroid Hormone-Responsive Protein (THRSP)–Overexpressing Mice
Source: Mol Neurobiol. 2025 Jun 10;62(10):13225–49. doi: 10.1007/s12035-025-05031-z (PMC12433356; doi:10.1007/s12035-025-05031-z)
Supplement: Supplementary file 6 — Supplementary Table 5 (DOCX 16 KB) [file 12035_2025_5031_MOESM6_ESM.docx]

Supplementary Table 5. List of RT-qPCR primers used for mRNA analysis.

| **Gene** | **Symbol** | **Primers** |
| --- | --- | --- |
| DENN domain-containing protein 4C | *dennd4c* | F: 5’-AGTGACAGACTACTTCGTTGTGG-3’ |
|  |  | R: 5’-GGAGCTTTAGGACCAATCGAGTT-3’ |
| Synaptosomal-associated protein 25 | *snap25* | F: 5’- CAACTGGAACGCATTGAGGAA-3’ |
|  |  | R: 5’-GGCCACTACTCCATCCTGATTAT-3’ |
| Dihydropyrimidinase-related protein 3 | *dpysl3* | F: 5’-CCGTCTTCTAATCAAGGGAGGG-3’ |
|  |  | R: 5’-AATCGTCCACTGTGGTCATTC-3’ |
| von Willebrand factor | *vwf* | F: 5’-CTTCTGTACGCCTCAGCTATG-3’ |
|  |  | R: 5’-GCCGTTGTAATTCCCACACAAG-3’ |
| Guanine nucleotide-binding protein G(i) subunit alpha-3 | *gnai3* | F: 5’-GAGCGGAGCAAGATGATCGAC-3’ |
|  |  | R: 5’-CGTCCTCTGAATAGCCGTCC-3’ |
| Syntaxin 1A | *stx1a* | F: 5’-AGAGATCCGGGGCTTTATTGA-3’ |
|  |  | R: 5’-AATGCTCTTTAGCTTGGAGCG-3’ |
| Syntaxin 1B | *stx1b* | F: 5’-CACTCCACACTCTCACGGAAG-3’ |
|  |  | R: 5’-TGCCTCTGGATTCGGTCCTT-3’ |
| Vesicle-associated membrane protein 2 | *vamp2* | F: 5’- GCTGGATGACCGTGCAGAT-3’ |
|  |  | R: 5’-GATGGCGCAGATCACTCCC-3’ |
| Synaptotagmin 1 | *syt1* | F: 5’-CTGTCACCACTGTTGCGAC-3’ |
|  |  | R: 5’-GGCAATGGGATTTTATGCAGTTC-3’ |
| Mammalian uncoordinated-13 | *munc13* | F: 5’-TGCTCTGTGTGCGTGTTAAAA-3’ |
|  |  | R: 5’-CAGACGACTGATCTCAAACATGA-3’ |
| Mammalian uncoordinated-18 | *munc18* | F: 5’- GTGGACCAGTTAAGCATGAGG-3’ |
|  |  | R: 5’-GCTCTCGGCGCTTGTTGAT-3’ |
| Calcium channel, voltage-dependent, beta 2 subunit | *cacnb2* | F: 5’-CATCACCCCACTCCAAAGAGA-3’ |
|  |  | R: 5’- CGCCCTTCAAATCTGTGTTTTAG-3’ |
| Calcium channel, voltage-dependent, beta 3 subunit | *dennd4c* | F: 5’-GGTTCAGCCGACTCCTACAC-3’ |
|  |  | R: 5’-GGGCACTCCTCATCCAGAA-3’ |
| Actin beta | *actb* | F: 5’-GGCTGTATTCCCCTCCATCG-3’ |
|  |  | R: 5’-CCAGTTGGTAACAATGCCATGT-3’ |
